# One-pot, degradable, silica nanocarriers with encapsulated oligonucleotides for mitochondrial specific targeting

**DOI:** 10.1186/s11671-023-03926-1

**Published:** 2023-12-21

**Authors:** Chloe Trayford, Alissa Wilhalm, Pamela Habibovic, Hubert Smeets, Florence van Tienen, Sabine van Rijt

**Affiliations:** 1 Department of Instructive Biomaterials Engineering, MERLN Institute for Technology-Inspired Regenerative Medicine, Maastricht University, P.O. Box 616, 6200 MD Maastricht, The Netherlands; 2 Department of Toxicogenomics, School for Mental Health and Neuroscience, Maastricht University, PO Box 616, 6200 MD Maastricht, The Netherlands

**Keywords:** Intracellular degradation, Mitochondria targeting, Hollow silica nanoparticles, Large pore silica nanoparticles, Anti-gene therapy

## Abstract

Mutations in nuclear and mitochondrial genes are responsible for severe chronic disorders such as mitochondrial myopathies. Gene therapy using antisense oligonucleotides is a promising strategy to treat mitochondrial DNA (mtDNA) diseases by blocking the replication of the mutated mtDNA. However, transport vehicles are needed for intracellular, mitochondria-specific transport of oligonucleotides. Nanoparticle (NP) based vectors such as large pore mesoporous silica nanoparticles (LP) often rely on surface complexation of oligonucleotides exposing them to nucleases and limiting mitochondria targeting and controlled release ability. In this work, stable, fluorescent, hollow silica nanoparticles (HSN) that encapsulate and protect oligonucleotides in the hollow core were synthesized by a facile one-pot procedure. Both rhodamine B isothiocyanate and bis[3-(triethoxysilyl)propyl]tetrasulfide were incorporated in the HSN matrix by co-condensation to enable cell tracing, intracellular-specific degradation and controlled oligonucleotide release. We also synthesized LP as a benchmark to compare the oligonucleotide loading and release efficacy of our HSN. Mitochondria targeting was enabled by NP functionalization with cationic, lipophilic Triphenylphosphine (TPP) and, for the first time a fusogenic liposome based carrier, previously reported under the name MITO-Porter. HSN exhibited high oligonucleotide incorporation ratios and release dependent on intracellular degradation. Further, MITO-Porter capping of our NP enabled delayed, glutathione (GSH) responsive oligonucleotide release and mitochondria targeting at the same efficiency as TPP functionalized NP. Overall, our NP are promising vectors for anti-gene therapy of mtDNA disease as well as many other monogenic disorders worldwide.

**Graphical Abstract:**

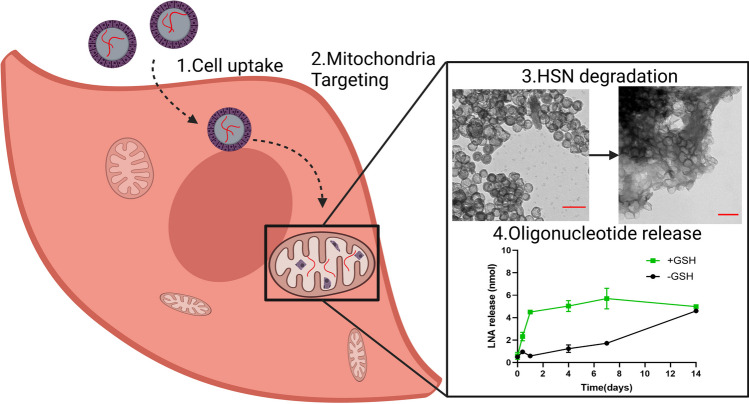

**Supplementary Information:**

The online version contains supplementary material available at 10.1186/s11671-023-03926-1.

## Introduction

Mitochondria provide the majority of cellular energy in the form of ATP via the oxidative phosphorylation (OXPHOS) system. Each mitochondrion contains multiple copies of mitochondrial DNA (mtDNA), which encode 13 OXPHOS subunits; these, together with nuclear-encoded proteins generate the OXPHOS system. A large number of pathogenic, heteroplasmic (mixture mutant and wild-type mtDNA) mutations have been identified in mtDNA that cause OXPHOS dysfunction and multisystem disorders (frequency 1/5000) [1]. Reducing mtDNA mutation load should prevent deterioration and establish clinical improvement but requires reaching the hundreds to thousands of mtDNA copies per cell that are located in the mitochondrial matrix and protected by a lipid bilayer. Strategies to reduce mtDNA mutation such as degrading mutant mtDNA using endonucleases or blocking mutant mtDNA replication with antisense oligonucleotides have been identified. However, enzymes and oligonucleotides are unable to cross membranes and are highly susceptible to degradation meaning *in-vivo* delivery to the mitochondrial matrix is challenging [2–4]. Mitochondrial delivery has been achieved using viral oligonucleotide carriers, e.g. adeno-associated viruses (AAV) but they are inherently unstable and subject to rejection [5, 6].

Nanoparticles (NP) represent an interesting alternative to viral vectors since they can be engineered to avoid immune system recognition and have high loading capacities due to their high surface area-to-volume ratio [7]. Several types of NP, including polymeric or liposomal nanocarriers, have been used for large biomolecule delivery, and show good biocompatibility and transfection efficiency, but mostly fall short in terms of (sub)cellular targeting, controlled cargo release, and tissue clearance ability [8].

Recently, large pore mesoporous silica nanoparticles (LP) have been developed as promising alternative vectors due to the high loading capacity and modification possibilities that have facilitated tracing and subcellular targeting. LP are formed from siloxane polymer and have large pore sizes of in between 6 and 50 nm that can be used for efficient oligonucleotide loading [9]. Commonly, LP are surface functionalized with amine groups by post grafting, which has enabled electrostatic complexation of negatively charged oligonucleotides on their surface [10, 11]. LP have been successfully implemented for the intracellular delivery of oligonucleotides in a range of therapeutic strategies including immunomodulation [12], chemotherapy [13] and stem cell differentiation [14, 15]. However, surface complexed oligonucleotides are prone to degradation lowering their efficacy, and additional surface modifications to enable e.g. improved nuclear [16] or mitochondrial [17] localization, are difficult to incorporate in the design. Finally, these systems do not allow for controlled release of oligonucleotides. These characteristics are especially important for oligonucleotide delivery to subcellular locations such as the mitochondria.

Hollow mesoporous silica nanoparticles (HSN) can circumvent some of these issues as a high quantity of oligonucleotides can be loaded in the tunable hollow central cavity protected from the external environment. In particular, HSN can be prepared using a reverse micro-emulsion system where water-in-oil droplets act as reaction centers for silica condensation. These HSN could encapsulate enzymes within the hollow core during the one-pot synthesis procedure [18, 19]. However, enzyme release was blocked by entrapment within the undegradable silica shell which has mesopores that are too small to allow release (< 2 nm). Thus, although HSN synthesized by reverse micro-emulsion show great potential for high efficiency in-situ core loading of biomolecules such as oligonucleotides, their inability to degrade hinders their use as gene therapy tools.

Here we developed, for the first time, fluorescent, biodegradable HSN with oligonucleotides encapsulated *in-situ* in the hollow core. To allow intracellular “on-demand” degradation of HSN, bis[3-(triethoxysilyl)propyl]tetrasulfide (BTES) was incorporated in the silica matrix by co-condensation allowing HSN to degrade in the presence of intracellular glutathione (GSH) by reduction. Additionally, to enable fluorescent tracing, rhodamine b isothiocyanate conjugated with 3-aminopropyl triethoxysilane (RITC-APTES) was also incorporated in the HSN matrix by co-condensation. As a comparison, also LP nanoparticles were synthesized and oligonucleotide uptake and release from both nanoparticles was investigated. Another advantage of HSN is that the surface can be easily modified to allow (sub)cellular targeting. Here, HSN and LP were surface modified with the mitochondrial targeting units TPP and, for the first time, a specific lipid composition termed MITO-Porter. A study by Y. Yamada et al. showed that this specific lipid composition led to the highest mitochondrial co-localization efficiency compared to a range of fusogenic lipids [20]. Here, we adapted MITO-Porter to absorb on the surface of our NP. We investigated the ability of both HSN and LP to target mitochondria in mesoangioblasts (MABs) isolated from patients with mtDNA mutations. MABs are muscle stem cells that can generate novel muscle fibres or fuse with existing muscle fibres, and that potentially allow reducing the mtDNA mutation load in both the MABs and muscles of the patient. Overall, here we created fluorescent, oligonucleotide loaded HSN and LP capable of mitochondria specific release, which represent promising gene delivery systems for the treatment of a myriad of mitochondrial disorders.

## Materials and methods

### Materials

Water was purified using a Milli-Q system at a conductivity of 18,2 MΩ cm^−1^ (Millipore, US). Iscove's Modified Dulbecco's Medium (IMDM), 2-mercaptoethanol, Minimum Essential Medium Non-Essential Amino-Acids (MEM-NEAA), insulin-transferin-selenium-X, gentamycin, phosphate-buffered saline (PBS) were purchased from gibco. MitoTracker Deep Red FM and PCR primers were purchased from Invitrogen. 2xSensiMix SYBR HI-ROX was purchased from meridiana. Triton X100, Rhodamine B isothiocyanate (RITC), 98% 3-aminopropyltriethoxysilane (APTES), cyclohexane, 1-hexanol, Igepal CA-630, Tetraethyl orthosilicate (TEOS), Bis[3-(triethoxysilyl)propyl]tetrasulfide (BTES), cetyltrimethyl ammonium bromide (CTAB), Triethylamine (TEA), ibuprofen, (3-Mercaptopropyl) trimethoxysilane (MPTES), ammonium nitrate (NH_4_NO_3_), hydrochloric acid (HCL, 37%), (4-Carboxybutyl)triphenylphosphonium bromide (TPP), N-Ethyl-N′-(3-dimethylaminopropyl) carbodiimide hydrochloride (EDC), N-hydroxysuccinimide (NHS), 1,2-dioleoyl-sn-glycero-3-phosphoethanolamine (DOPE), sphingomyelin (SM), chloroform, sodium cacodylate, osmium tetroxide, Potassium hexacyanoferrate, propylene oxide, 2,4,6-Tris(dimethylaminomethyl)phenol (DMP-30), 100% Ethanol DNA and LNA/DNA was purchased from Sigma-Aldrich. PNA was purchased from Integrated DNA technologies EU (IDT BVBA, Leuven, BE). The oligonucleotide sequences are as follows; DNA, GGG TTT GGT AAG ATG GCA GG-C3, LNA/DNA, GG[+ G] TT[+ T] GG[+ T] AA[+ G] AT[+ G] GC[+ A] GG and PNA, TGG CAG GGC CCG with mutation site indicated in Table S1. Bovine serum albumin (BSA), aqueous ammonia (28-30wt%), and paraformaldehyde (PFA) were purchased from VWR. F96 MicroWell Black Polystrene Plate, Hoechst 33,342, Lab-Tek II Chamber Slide System and phalloidin Alexa Fluor 647 were purchased from Thermo Fisher Scientific. Stearyloctaarginine (STR-R8) was purchased from LifeTeIn. Fetal bovine serum (FBS) was bought from Bodingco B.V. Uranyl acetate was bought from Agar scientific Ltd. Karnovskys fixative was purchased from Science Services. Epon 828 epoxy resin was purchased from Silmid (UK).

### Cell culture and oligonucleotide testing

Mesoangioblasts (MABs) with a m.3243A > G mutation load of 80% were previously isolated from a skeletal muscle biopsy of patient with mitochondrial myopathy and were cultivated in IMDM with 10% FBS, 0.2% 2-mercaptoethanol, 1% MEM NEAA, 1% insulin-transferrin-selenium-X, 0.01% FGF2 and 0.1% gentamycin at 37 °C 5%CO2/4%O2 according to Tonlorenzi et al. [21, 22].

### HSN synthesis and oligonucleotide incorporation

HSN with incorporated RITC and BTES were synthesized via adaption of a previously reported reverse micro-emulsion system [23]. First, RITC-APTES was prepared by reacting 5 mg RITC with 44 µL of APTES (molar ratio RITC: APTES = 1:10) in 1 mL of ethanol and stirred overnight in the dark. Next, a reverse micro-emulsion system was made with cyclohexane (19.06 mL, 176 mmol) Igepal CA-630 (5 g, 15 mmol) and n-hexanol (3 mL, 24 mmol) and stirred for 10 min in a round bottom flask. Then, in a 100 µL mixture of TEOS/BTES (v/v = 5:3) and added into the emulsion mixture along with RITC-APTES (25 µL, 0.0023 mmol) and 350 µL water. For HSN synthesis with incorporated oligonucleotides or HSN(LNA)s, 50 µM of oligonucleotide was used instead of water. After stirring the emulsion in the dark for 2 h, 250 µL of ammonia (28–30 wt%) was added and kept at RT under vigorous stirring for 36 h. To isolate the silica nanospheres 80 mL ethanol was added and product was collected by centrifugation (7745 × g, 25 min, 10 °C). The NP were washed twice in ethanol to remove excess surfactant. Then to remove the hollow core, solid nanospheres were stirred in 100 mL water at 50 °C overnight. HSN were then collected by centrifugation (7745 × g, 25 min, 10 °C) and the pellet finally dispersed in a 25 mL mixture of ethanol/water (v/v = 70/30). HSN were then stored as a suspension in 70% ethanol at − 20 °C for up to 6 months. Before use, HSN(LNA) were washed twice with sterile water, and HSN(LNA) pellet was re-suspended in PBS or cell culture media and immediately used in further experiments.

### LP synthesis, post grafting and LNA incorporation

LP with amines grafted on the surface and with RITC incorporated were synthesized by a modified Stöber method using ibuprofen as an auxiliary template [24]. First TEA (400 mg, 4 mmol) was added to 20 mL water at 80 °C under reflux and vigorous stirring for 30 min. Then, CTAB (304 mg, 0.83 mmol) was added to the solution and stirred for 1 h before ibuprofen (69.9 mg, 33.9 mmol) was added. The mixture was stirred for a further 3 h to ensure the incorporation of ibuprofen within CTAB micelles. Then, TEOS (3.2 mL, 2.99 mmol) and RITC-APTES (250 µL, 0.023 mmol) was added and the solution was stirred for 20 min. The resulting LP were collected by centrifugation (7745 × g, 15 min) and the precipitate was redispersed using a sonicator (BRANSON 3800 DTH, US) which was used for all washing steps. The LP were then washed twice with ethanol to remove residual reactants. Next, CTAB was removed by extraction using ammonium nitrate solution in ethanol (10 wt%, 100 mL) for 45 min under reflux at 90 °C. An additional washing was performed using HCl in ethanol (1%, 100 mL) under the same conditions as for the initial extraction step. The resulting LP were then washed twice with ethanol by centrifugation (7745 × g, 15 min) and stored at a concentration of 10 mg/mL in ethanol at − 20 °C. For post-grafting -NH_2_ onto the LP surface, 50 mg of LP were dispersed in 50 mL ethanol under reflux at 90 °C. Then, APTES (500 µL, 0.38 mmol) was added under continuous stirring and left to react overnight. LP were collected by centrifugation (7745 × g, 25 min, 10 °C), washed with ethanol three times and similarly to LP at a concentration of 10 mg/mL in ethanol at − 20 °C. LP_PG_ were loaded with LNA by dispersing 1 mg in 200 µL of 50 µM LNA overnight. LP(LNA) were washed twice with sterile water to remove unloaded LNA. The LP(LNA) pellet was immediately used in further experiments by re-suspending in PBS or cell culture media.

### HSN and LP mitochondrial targeting surface modification

HSN and LP were either conjugated to TPP (HSN_TPP_, LP_TPP_) or coated with ‘MITO-Porter’ (HSN_M_, LP_M_) for mitochondrial targeting. MITO-porter consisted of DOPE, SM, and STR-R8 in a molar ratio of 9:2:2, which has been shown to promote mitochondrial targeting [20]. For LP(LNA)_M,_ LNA was loaded prior to MITO-Porter functionalization and for LP(LNA)_TPP_ LNA was loaded after. For TPP functionalization, TPP (50 mg, 0.08 mmol) was dissolved in 5 mL PBS at pH 7. Then, EDC (25 mg, 0.16 mmol) and NHS (35 mg, 0.3 mmol) was added and the solution was stirred for 2 h in the dark to activate the carboxylic acid groups of TPP. For NP attachment 2 mg of HSN or LP were dissolved in 200 µL water, added to the activated TPP solution and stirred overnight at RT in the dark. To remove unreacted TPP, NP were washed three times in ethanol using centrifugation (30,130 × g, 5 min). To functionalize the surface of our NP with MITO-Porter the hydration method was used [25]. Specifically, DOPE (3.08 mg), STR-R8 (1.42 mg) and SM (1.36 mg) were dissolved in chloroform at the molar ratio of 9:2:2. Chloroform was then evaporated from the solution by rotary evaporation (Neuberger, US) until a lipid film formed. To ensure the complete removal of chloroform, the lipid film was dried overnight at 40 °C. The film was then dissolved in an ethanol/ water mixture (40/60; v/v) to obtain a 2.5 mg/mL lipid solution. For NP functionalization, 100 µL was added to 1 mg of NP and sonicated. Then, 700 µL water was added and the solution was incubated for 10 min to form a lipid bilayer termed ‘MITO-Porter’ over the NP. Then, the excess lipids were removed by centrifugation (30,130 × g, 5 min) and the pellet was washed twice with water.

### HSN and LP characterization

Morphological characterization was performed by transmission electron microscopy (TEM) using a FEI Tecnai electron microscope. For imaging, NP suspensions (0.3 mg/mL, 5 µL) were spotted on a 200 mesh carbon grid and imaged after air drying at RT overnight. To visualize MITO-Porter, grids were stained with 1% aqueous UA for 30 min. For NP size analysis, the particle analysis function on ImageJ was used. Electrokinetic potential (ζ) was measured using the Malvern Zetasizer Nano (Malvern Panalytical, UK) at 25 °C at an angle of 90° and NP were suspended in water at a concentration of 0.3 μg/mL. To minimize pH effects, we used the same batch of MQ water and verified the pH (7) prior to each NP dispersion and measurement. To confirm post-grafting and surface modification, zeta potential was read and compared to LP and HSN without functionalization. To assess the RITC and LNA concentration of LP and HSN, RITC and LNA-FAM standard curves were made from 0 to 2.5 µM. Fluorescence quantifications were performed using a CLARIOstar spectrophotometer equipped with MARS data analysis software (BMG LABTECH, Germany) measuring standard curves together with particles in triplicate at 100 µg/mL in a 96 wp. The fluorescent signal for LNA-FAM was detected at *λ*_ex_ = 488 nm and *λ*_em_ = 521 nm and for RITC at *λ*_ex_ = 560 nm and *λ*_em_ = 595 nm.

### HSN(LNA) and LP(LNA) release and degradation

The release kinetics of LNA from LP(LNA) or HSN(LNA) was assessed by measuring LNA concentration in the supernatant of solvated samples at different timepoints. First, 2 mg of LNA incorporated NPs were dispersed in 2 mL PBS or PBS containing 3 mM GSH. The samples were incubated in a ThermoMixer® C (Eppendorf, GE) (37 °C, 500 rpm). At the time points; 0 h, 6 h, 1 d, 4 d, 7 d, and 14 d, 100 µL of solution (in triplicate) was removed from each sample and the NPs were collected by centrifugation (30,130 × g, 5 min, RT). The supernatant was removed and the oligonucleotide concentration was measured using a Nanodrop (Witec AG, CH). Then, collected pellets were additionally resuspended in PBS and spotted for TEM (Sect. 2.6).

### HSN(LNA) and LP(LNA) biocompatibility and cell uptake

An MTS assay was performed to assess cell metabolism after LP_TPP_, LP_M_, HSN_TPP_ and HSN_M_ uptake. For imaging, MABs medium consisted of Gibco DMEM/F12 HEPES no phenol red instead of IMDM medium with phenol red. MABs cells were exposed to LP_TPP_, LP_M_, HSN_TPP_ and HSN_M_ for 24 h in concentrations 0–500 µg/mL at 60–80% confluency in triplicate. Controls consisted of unexposed cells. For both assays, the medium was aspirated and the cells were washed twice with PBS before adding assay reagents. For the MTS assay, 80 µL of fresh imaging medium as well as 20 µL of MTS/PMS solution (2 mg/mL/ 0.92 mg/mL, 20:1 v/v) was added to each well and incubated for 3 h in a 5% CO_2_ incubator at 37 °C and absorbance was read at 490 nm using a CLARIOstar spectrophotometer equipped with MARS data analysis software (BMG LABTECH, Germany) as previously described [26]. Flow cytometry (BD Accuri C6) was performed to quantitatively investigate the cellular uptake of HSN_M_, HSN_TPP_, LP_M_ and LP_TPP_ after 24 h incubation. Therefore, MABs were seeded in 12 well plates at 5000 cells/cm^2^ and exposed to 100 µg/mL of NPs for 24 h. To prepare MABs for flow cytometry, the cells were washed with PBS, trypsinized and resuspended in 300 µL PBS on ice. For each measurement, 10,000 cells were collected and control cells were used to gate the cells accordingly. FlowJo (FlowJo V10, LLC) was used for data analysis.

### HSN and LP mitochondrial targeting

Live cell imaging of MABs was conducted to investigate the co-localization of our NPs with mitochondria. MABs were seeded in an 8-well chamber slide and incubated with 100 μg/mL of HSN, LP, HSN_M_, LP_M_, HSN_TPP,_ and LP_TPP_ for 24 h. The medium was aspirated, washed twice with PBS, and the nuclei were stained with Hoechst (1 μg/mL) for 5 min at 37 °C. The cells were washed again with PBS and stained with Calcein AM (10 μM) for 30 min at 37 °C. Then the cells were washed again with PBS and stained with MitoTracker deep red (100 nM) for an additional 30 min at 37 °C. Just before imaging, the cells were washed with PBS and replaced with imaging media. An automated inverted fluorescence microscope (Nikon Ti-E), equipped with a Lumencor Spectra X light source, Photometrics Prime 95B sCMOS camera, MCL NANO Z500-N TI z-stage, and an Okolab incubator (37 °C, 5% CO_2_) was used for image acquisition as previously described [26]. Excitation was set to 390 nm (Hoechst), 488 nm (Calcein-AM), 567 nm (NPs), and 647 nm (Mitotracker). Mitochondrial targeting analysis was performed in NIS Element 5.30.01 using the GA3 analysis module. Background subtraction using a rolling ball was performed, after which MABs were thresholded based on the Calcein signal and segmented using “separate objects”. To prevent the detection of cell remnants, MABs were only included in the analysis if they contained a single nucleus, which was thresholded separately based on DAPI. Cells touching the border of the frame were excluded from the analysis. Subsequently, the Pearson’s correlation coefficient comparing Mitotracker to NP signal was measured in individual MABs of 6 fluorescence images. We also performed TEM sectioning to investigate cell and mitochondria uptake of NPs at higher resolution. Mutated MABs were seeded in a 6-well cell culture plate until 90% confluency before incubation with NP. After 24 h of incubation with NP, the cell medium was removed, and the cells incubated with Karnovskys fixative for 1 h at RT, then for 2 d at 4 °C. Then, the cells were washed four times for 5 min with Sodium Cacodylate buffer (0.1 M, pH 7.4) at RT. Afterwards, the cells were postfixed for 1 h in the dark with osmium tetroxide(1%), potassium hexacyanoferrate (1.5%) in sodium cacodylate buffer (0.065 M, pH 7.4). The post-fixed cells were then washed with water until the solution ran clear. Next, the samples were dehydrated at RT for 30 min each round twice in 70% EtOH, three times in 90% EtOH and 100% EtOH, and finally three times in propylene oxide. Finally to embed MABs for TEM imaging, three 1 h cycles of embedding were conducted using DMP-30 as the accelerator and increasing the percentage of epon in each round. First resin (Epon: PO, v/v = 1:3 + 2% DMP-30) was used, then 2:2 in the second cycle and 3:1 in the third. Finally, the samples were covered with pure Epon and incubated at 60 °C for 3 days.

### Statistics

Results are expressed as a mean ± SD (standard deviation). Statistical analysis was performed using GraphPad PRISM (GraphPad Software, USA). One-way and two-way ANOVAs were used for comparison among groups. To compare LNA release kinetics from HSN(LNA)_TPP_ the Higuchi theoretical model was used; Q = K_H_t^1/2^ [27]. Therefore, when our LNA release data are plotted as cumulative release (Q) vs square root of time (h) the slope of the plot (K_H_) refers to the rate constant of LNA diffusion through the silica shell of HSN(LNA). Results were considered statistically significant at *p* < 0.1

## Results and discussion

### Synthesis and surface modification of HSN and LP

**Fig. 1:**
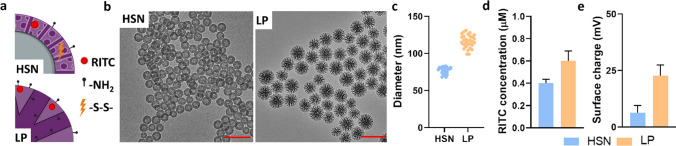
HSN and LP_PG_ synthesis and characterization. **a** Schematic of HSN and LP_PG_ where RITC refers to RITC-APTES,–NH_2_ to APTES, and –S–S- to BTES. **b** TEM images of HSN and LP_PG_. Scale bars are 200 nm. **c** Size analysis of 30 particles by Image J where HSN = blue and LP_PG_ = orange **d** Concentration of RITC in 100 µg of NP as determined by fluorescence analysis where HSN = blue and LP_PG_ = orange. **e** Zeta potential analysis of HSN and LP_PG_ where HSN = blue, LP_PG_ = orange

To develop the degradable HSN for mitochondrial-targeted oligonucleotide delivery, first HSN were synthesized using a reverse micro-emulsion method [28], and co-condensed with BTES to allow intracellular oligonucleotide release (Fig. 1a- top). BTES is a disulfide bridged siloxane that is reduced in GSH rich intracellular environments. Moreover, to enable NP tracing, a fluorescent dye-siloxane conjugate, RITC-APTES, was incorporated in the matrix of HSN by co-condensation. Synthesized HSN were uniform with a round morphology, hollow core, and mesopores as observed by TEM (Fig. 1b- left). Image J analysis of HSN revealed a diameter of 77 ± 4 nm (Fig. 1c). 0.4 ± 0.1 µM RITC was incorporated in the matrix of 100 µg/mL HSN which had a surface charge of 6.3 ± 3.2 mV, likely due to the presence of positive RITC-APTES on the surface (Fig. 1d-e, S1). Of note, all nanoparticle suspensions were measured in miliQ water with pH 7, this, however, does not account for differences in ionic strength of the nanoparticle suspensions, and may cause some variability in the zeta measurements. Thus, here we developed highly fluorescent HSNs with incorporated BTES and RITC. Several groups have reported etched-HSN with incorporated BTES for intracellular specific degradation [29, 30] however, we are the first to demonstrate HSN with incorporated BTES and RITC synthesized by reverse micro-emulsion.

LP reference NPs were synthesized by a modified dual-template stöber approach using ibuprofen as an auxiliary template and with incorporated RITC-APTES (Fig. 1a- bottom) [31]. To increase surface charge and improve oligonucleotide loading, amine groups were added at the surface by post-grafting (*ex-situ* surface modification) or by co-condensation with APTES ((3-Aminopropyl)triethoxysilane). The surface charge of post-grafted LP increased to 31.6 ± 12.6 mV, while co-condensation resulted in LP with a surface charge of 8.9 ± 4.9 mV (LP_D_, Figure S2a). Although both LP were significantly more positive than LP without amine modification (LP_C_; − 0.1 ± 5.6 mV, *p* < 0.0001, Fig. 1e, S2a), post-grafted LP were selected for further studies due to their higher surface charge. LP were spherical with dendritic large pores, a diameter of 116 ± 9 nm (Fig. 1b-c), and 0.6 ± 0.1 µM RITC per 100 µg/mL NP (Fig. 1d, S1).

We further investigated whether we could incorporate thiol groups in the LP core first by co-condensation with MPTES ((3-Mercaptopropyl)trimethoxysilane) and then post-grafting with APTES to create LP_SH._ The resulting NP were positive (23.2 ± 4.7 mV, *p* < 0.0001, Figure S2a) and the presence of the amine and thiol groups was confirmed by reaction with a mixture of amine reactive FITC and thiol reactive ATTO-647 fluorescent dyes, forming tri-fluorescent LP_SH_ (Figure S2b).

Although LP have been actively researched for over a decade as efficient silica-based drug delivery systems [32, 33], we are the first to describe LP with a RITC-modified matrix and orthogonal surface (-NH_2_) and pore (-SH) functionalization. These LP can be functionalized both at the free amine and thiol groups using a variety of probes (e.g. fluorescent dyes, therapeutics, sensors) while the matrix remains fluorescent for real-time tracing (Figure S2b). This is a promising strategy to impart further functions to the NP such as (sub)cellular targeting, sensing, or imaging multimodality [34]. Overall, we successfully synthesized multifunctional LP and HSN.

### Oligonucleotide incorporation and surface modification of HSN and LP

**Fig. 2:**
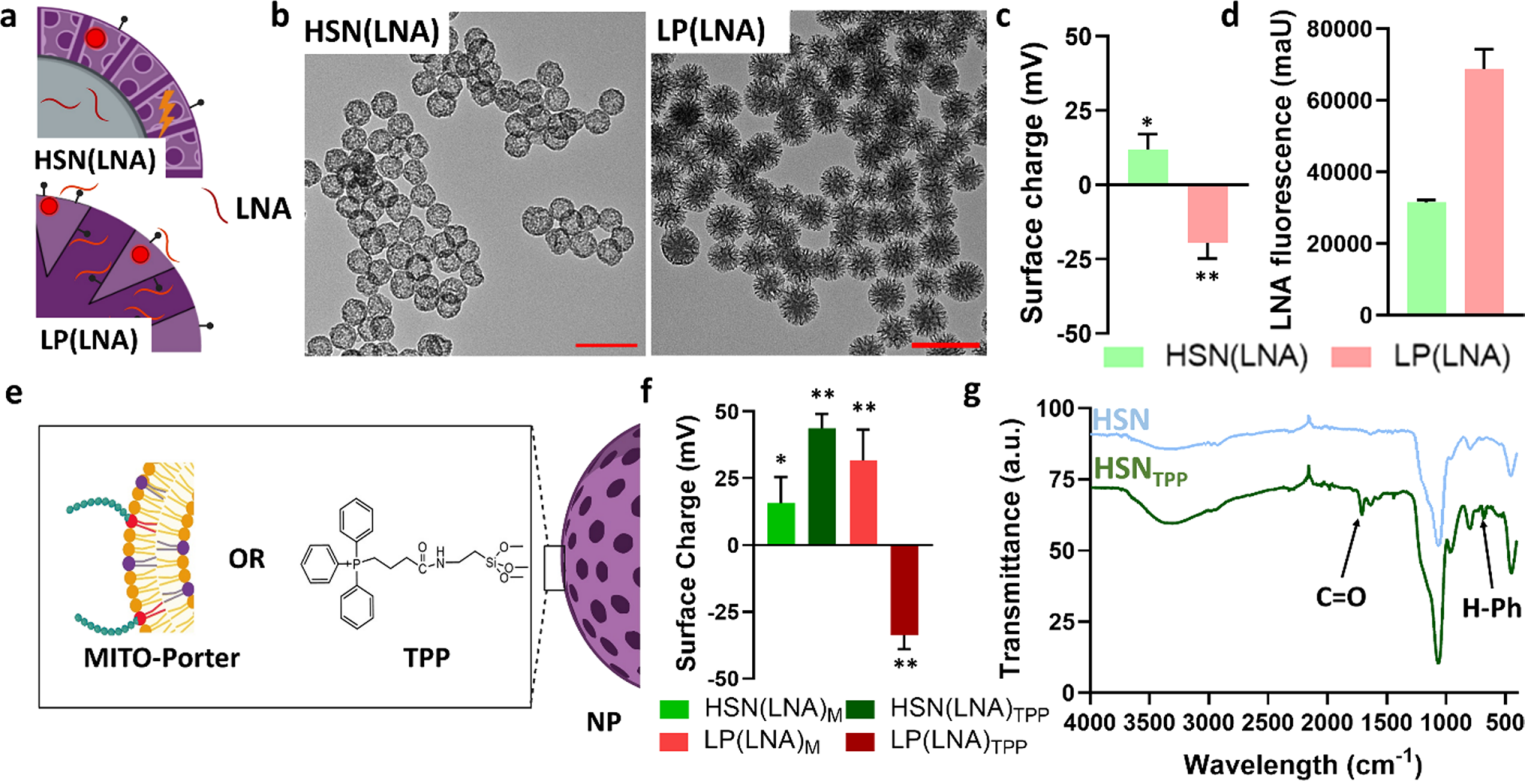
HSN(LNA) and LP(LNA) synthesis, surface modification, and characterization. **a** Schematic of HSN(LNA) and LP(LNA). **b** TEM images of HSN(LNA) and LP(LNA) where scale bars are 200 nm. c Surface charge of NP where HSN(LNA) = light green and LP(LNA) = pink. Statistical significance is determined compared to the surface charge of HSN and LP. **d** LNA-FAM content per mg of NP by fluorescence analysis. **e** Schematic of surface modification of NP with MITO-Porter or TPP. **f** Surface charge of NP after LNA incorporation and surface modification by zeta potential analysis where HSN(LNA)_M_ = green, HSN(LNA)_TPP_ = dark green, LP(LNA)_M_ = red, and LP(LNA)_TPP_ = dark red. Statistical relevance is determined compared to HSN(LNA) and LP(LNA) **g** FTIR spectroscopy showing emerging bond vibrations attributed to H-Ph of TPP and C = O of amide linkage. Significance is shown as * = p < 0.01 and ** = p < 0.0001

Next, oligonucleotides (LNA/DNA, DNA, PNA) were incorporated in HSN and LP nanoparticles (Fig. 2a, Table S1). To create HSN(LNA), a 33%LNA/67%DNA solution was added to the bulk oil phase, forming aqueous droplets of the reverse micro-emulsion, which were reaction centers for silica condensation (Fig. 2a- top). The encapsulation of different types of oligonucleotides was also investigated by replacing LNA/DNA solution with a DNA or PNA solution. We found that HSN were able to form in the presence of LNA/DNA (Fig. 2b-left) or DNA (Figure S3a) but not PNA (Figure S3b). The diameter or RITC signal of HSN(LNA) did not change compared to HSN (Figure S4, S1). However, the surface charge of HSN(LNA) was significantly higher than HSN (11.8 ± 5.3 mV, *p* < 0.01, Fig. 2c), possibly due to encapsulation of LNA/DNA causing positively charged RITC-APTES to migrate to HSN perimeters. The presence of LNA was confirmed by synthesis of HSN(LNA) with FITC-labelled LNA (LNA-FAM) resulting in green fluorescent NP (Fig. 2d). To confirm the encapsulation of LNA in the hollow core of HSN, LNA-FAM fluorescence and surface charge of HSN(LNA) was measured after washing 3 times. We observed that neither the fluorescence nor surface charge significantly changed (Figure S5). HSN have been developed as gene carrier systems in several studies [35–37]. However, in these approaches, oligonucleotides were complexed at the surface and not encapsulated in the hollow core, as we have done here. Our approach has the advantage that the HSN silica shell protects encapsulated oligonucleotides from degradation and enables HSN to be stored without the threat of oligonucleotide leakage.

To form LP(LNA), LP were immersed in LNA solution overnight, enabling active LNA loading in the pores by electrostatic absorption (Fig. 2a- bottom). Similarly to HSN, LNA incorporation in LP did not affect morphology, size, or RITC signal (Fig. 2b- right). However, in contrast to HSN(LNA), a drastic decrease in surface charge was observed from 31.6 ± 12.6 mV for LP to − 19.5 ± 5.3 mV for LP(LNA) due to absorbed negative LNA masking the positively charged LP surface (*p* < 0.0001, Fig. 2c). Further, LNA-FAM absorption was highly dependent on NP type and surface charge; ~ twofold more LNA-FAM was incorporated in LP(LNA) compared to HSN(LNA), while LP_C_ (LP with incorporated RITC but without amine post grafting) absorbed approximately tenfold less than LP (Fig. 2d, S6). The higher LNA incorporation in LP compared to HSN, is likely due to the increased surface charge and area of LP, allowing higher amounts of LNA to adsorb (Fig. 2d). Thus, surface charge and area are important factors to consider when designing NP for oligonucleotide delivery.

To facilitate intra-mitochondrial delivery, the surface of HSN and LP were modified with two different mitochondrial targeting moieties, MITO-Porter and TPP (Fig. 2e). MITO-Porter is composed of DOPE, SM, and STR-R8 in a molar ratio of 9:2:2 (Fig. 2e- orange, purple and red, respectively), which provide a positive charge and lipophilicity to our NPs as well as enable cell uptake and endosomal escape [20]. Both TPP and MITO-Porter enable mitochondrial membrane fusion and accumulation [38]. TPP was conjugated to the surface of HSN and LP via an amide bond to create HSN_TPP_ and LP_TPP_ while MITO-Porter was surface adsorbed by the hydration method forming HSN_M_ and LP_M_. LNA incorporated HSN and LP were also surface modified with TPP and MITO-Porter, to form HSN(LNA)_TPP_, HSN(LNA)_M_, LP(LNA)_TPP,_ and LP(LNA)_M_. Due to the highly positive nature of MITO-Porter and TPP, the surface charge of HSN_M_ and HSN_TPP_ was significantly higher compared to non-modified HSN (25.6 ± 5.2 for HSN_M_ and 46.3 ± 9.0 mV for HSN_TPP_) (p < 0.0001, Figure S7). This was also observed for HSN(LNA) with surface charges increasing to 15.6 ± 9.8 mV and 43.7 ± 5.4 mV for HSN(LNA)_M_ and HSN(LNA)_TPP_, respectively (*p* < 0.0001, Fig. 2f). Similarly, TPP modification of LP also increased the surface charge (48.8 ± 11.8 mV, *p* < 0.0001, Figure S7). However, due to surface absorption of negative LNA, LP_TPP_ surface charge drastically decreased (− 33.8 ± 5.2 mV, Fig. 2f). Further, MITO-Porter functionalization of LP did not affect the surface charge, while for LP(LNA), a drastic increase was observed to 31.6 ± 11.6 mV (Figure S7, 2f). To further confirm the presence of MITO-Porter lipid bilayer, fluorescence analysis, and TEM imaging were used. For fluorescence analysis of HSN_M_ and LP_M,_ 2 mol% DOPE was replaced with the fluorescent lipid 1,2-dioleoyl-*sn*-glycero-3-phosphoethanolamine (18:1 (Δ9-Cis) PE) and co-localization of 18:1 (Δ9-Cis) PE signal at 488 nm and NP at 567 nm was observed (Figure S8). Moreover, TEM imaging revealed that the hollow core of HSN_M_ was less visible and LP_M_ reduced in porosity (Figure S9). To confirm TPP functionalization, Fourier-transform infrared spectroscopy (FTIR) was used. FTIR analysis of HSN_TPP_ and LP_TPP_ revealed emerging peaks at 700 cm^−1^ and 1650 cm^−1^ corresponding to deformation and stretching vibrations of phenyl C–H groups and amide C = O of TPP (Fig. 2g). The same peaks were also observed for LP_TPP_ (Figure S10).

Here we showed that LP and HSN could be successfully surface functionalized with TPP and MITO-Porter for mitochondrial targeting. Although several reports show MSN surface functionalization with lipid bilayers to improve colloidal stability [39] and reduce cargo leakage [40], to the best of our knowledge, there are no reports on NPs functionalized with MITO-Porter lipids.

### LNA release and HSN degradation

**Fig. 3:**
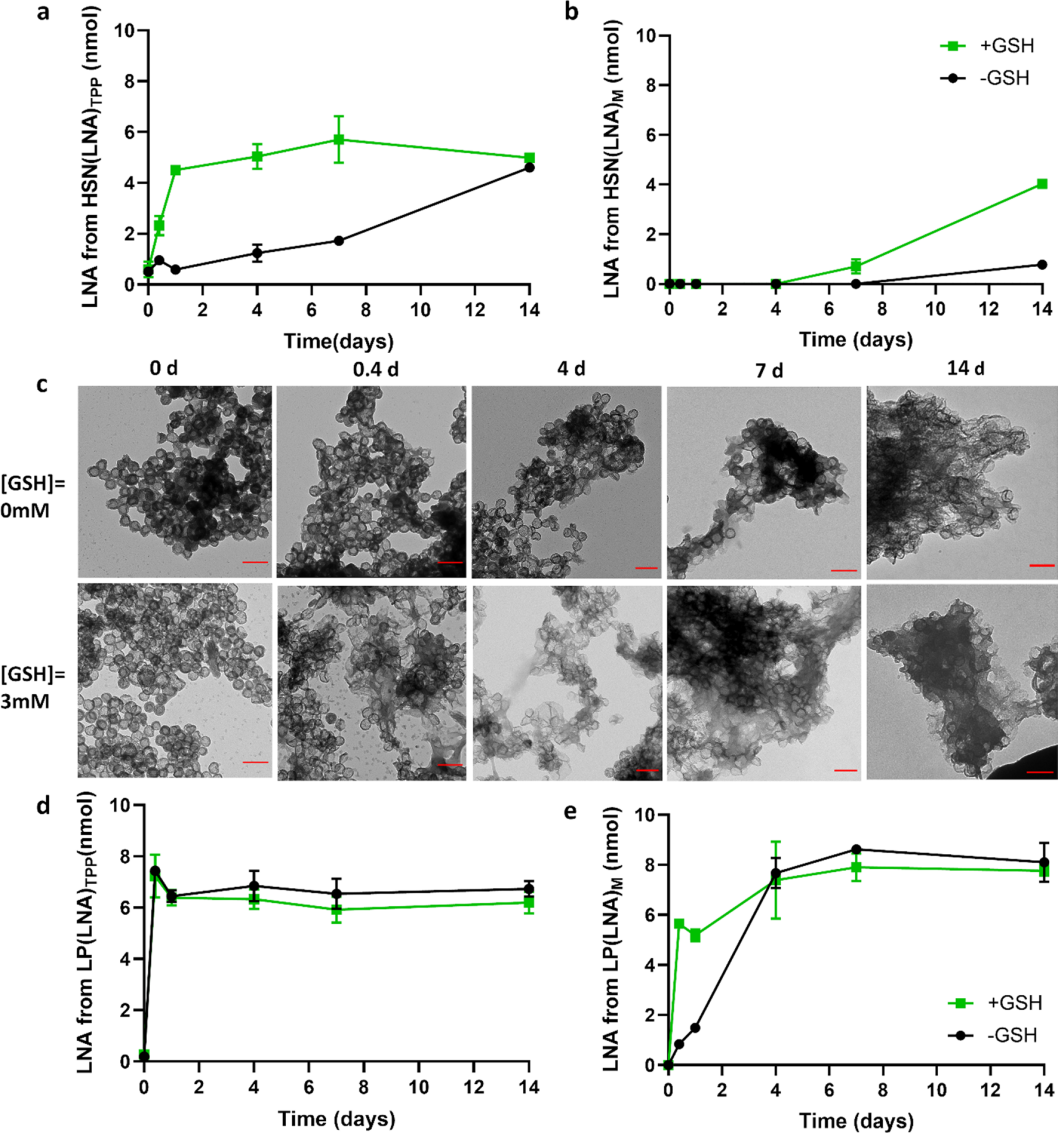
Release kinetics of LNA from HSN(LNA)_TPP_, HSN(LNA)_M_, LP(LNA)_TPP,_ and LP(LNA)_M_ and degradation analysis of HSN(LNA) by TEM. For release kinetics, NP were dispersed in PBS with either 0 mM or 3 mM GSH and the LNA concentration was measured by UV–Vis over 14 days for **a** HSN(LNA)_TPP_, **b** HSN(LNA)_M_. Error bars are derived from triplicates. **c** TEM images showing the GSH-catalyzed degradation of the collected HSN(LNA)_TPP_ pellets over 14 days. Scale bars are 200 nm. LNA release kinetics by UV–Vis for **d** LP(LNA)_TPP_ and **e** LP(LNA)_M_ over 14 days

Next, LNA release profiles of HSN(LNA)_TPP_, HSN(LNA)_M_, LP(LNA)_TPP,_ and LP(LNA)_M_ in PBS or in 3 mM GSH (to mimic the intracellular GSH-rich environment) were measured over 14 days (Fig. 3a-b, d-e). Both HSN(LNA)_TPP_ and HSN(LNA)_M_ displayed faster LNA release when GSH was present. Specifically, 1 mg of HSN(LNA)_TPP_ displayed gradual time-dependent LNA release from 0.6 ± 0.3 nmol at 0 days increasing at each time point to a maximum release of 5.0 ± 0.1 nmol at 7 days (*p* < 0.0001, Fig. 3a). However, when HSN(LNA)_TPP_ was dispersed in PBS, LNA release was fourfold less after 4 days compared to the release observed in the presence of GSH. LNA release kinetics from HSN(LNA), can be analyzed through an equation described by Higuchi et al., which takes into account the impact of the silica shell as a diffusion barrier [27, 41]. The Higuchi model is defined by the expression Q = K_H_t^1/2^, where K_H_ is the Higuchi dissolution constant and refers to the rate constant of LNA diffusion through the silica shell. The calculated K_H_ of LNA from HSN(LNA)_TPP_ in PBS was 0.6, while K_H_ in the presence of GSH was 4 times higher (K_H_ = 2.4, Figure S11). Interestingly, for HSN(LNA)_M,_ LNA release only began after 7 days in the presence of GSH and 14 days in neutral conditions, indicating that MITO-Porter functionalization is capable of delaying LNA release by up to two weeks. By TEM analysis, we also found that HSN(LNA)_TPP_ and HSN(LNA)_M_ exhibited GSH-dependent degradation (Fig. 3c, S12), which could be correlated to the respective LNA release profiles (Fig. 3a-b, S11).

Overall, LP(LNA)_TPP_ and LP(LNA)_M_ exhibited faster LNA release than HSN(LNA). For LP(LNA)_TPP_ maximum LNA release was already observed after 6 h reaching 6.7 ± 0.2 nmol in PBS which was indistinct from release in the presence of GSH (Fig. 3d). While for LP(LNA)_M_ the release rate was 3.5 X faster in the presence of GSH and reached a maximum of 7.9 ± 0.4 nmol after 7 days (*p* < 0.0001, Fig. 3e) indicating that MITO-Porter might be degraded by GSH. These findings suggest that MITO-Porter is both responsive to GSH and acts as a gating system for LNA release from our HSN and LP.

Here we showed that both HSN(LNA) and LP(LNA) can efficiently release high amounts of LNA. Interestingly, despite being commonly used for gene delivery studies, oligonucleotide release kinetics from LP and HSN have not previously been reported, only oligonucleotide incorporation efficiencies were measured which were between 0.4 and 9 nmol oligonucleotides/mg [11, 32, 33, 35, 37, 42, 43]. In these studies, NP with relatively low oligonucleotide incorporation demonstrated transfection efficiencies comparable to established gene vectors such as Oligofectamine or Fugene [44]. For example, Zhan et al. described HSN with 0.8 nmol/mg pDNA transfected LoVo cells at efficiencies up to 48% [35]. While for LP with 2.5 nmol/mg siRNA a ~ 30% higher reduction in cancer cell viability was observed compared to oligofectamine [11]. Therefore, our HSN(LNA) and LP(LNA) that release up to 5.0 ± 0.1 nmol and 8.6 ± 0.1 nmol LNA/mg, respectively, should in theory, demonstrate comparable efficacy to what has previously been observed.

Further, we showed that HSN(LNA) and LP(LNA) display different release profiles depending on the presence or absence of GSH. Specifically, LP(LNA) exhibited fast LNA release throughout 6 h, indicating that quick NP uptake into the cell (< 6 h) is needed for their optimal use. Thus, LP could be used in endothelial or cancer cells that display NP uptake < 6 h [45, 46]. In contrast, slow LNA release was observed from HSN(LNA) probably because the release was dependent on silica sphere degradation (Fig. 3a-c, S12). Co-condensation of BTES in HSN allows oligonucleotides to be released upon degradation in intracellular conditions. This eliminates the need for laborious post-synthetic loading and gating modifications that are necessary for controlled HSN gene delivery [36, 37]. Further, degradation of HSN enables cell elimination and clearance preventing side effects of NP accumulation, such as inflammation [47]. Significantly reduced HSN(LNA) degradation and cumulative release was observed in the absence of GSH, due to the lack of reduction of disulfide bonds in the HSN matrix. These results indicate that HSN(LNA) are relatively stable in extracellular spaces, lowering possible associated risks with premature, extracellular oligonucleotide release. Additionally, several studies report the use of GSH responsive etched-HSN to deliver drugs, such as DOX [48, 49]. However, in these HSN only small molecules could be delivered, which were released within 1 day. Sustained, intracellular release of oligonucleotides as in our HSN(LNA) can help maintain an effective therapeutic dose in cells increasing efficacy and mitigating possible negative side effects [50]. For example, the gradual release of antisense oligonucleotides comparable to mtDNA turnover (~ 2 weeks) reduces the risk of homeostatic imbalance and cell death caused by blocking the replication of too many mtDNA [51].

We also found that MITO-Porter surface functionalization of LP(LNA) and HSN(LNA) resulted in delayed LNA release of 4 and 14 days, respectively. Delayed LNA release by MITO-Porter functionalization could be a result of the charge stabilization of LNA with highly positive STR-R8 and steric hindrance of LNA passing through the lipid layer. Previous reports of lipid bilayer functionalized MSN showed delayed cargo release up to 48 h [26, 52–54]. Moreover, MITO-Porter functionalization of HSN(LNA) also showed reduced degradation (Figure S11) [55]. We also observed that the rate of LNA release from LP(LNA)_M_ was significantly increased in the presence of GSH. This is likely due to the degradation of MITO-Porter in the intracellular environment as also observed for similar liposome formulations [56]. Thus, MITO-Porter functionalization can be used as a novel tool for introducing intracellular responsivity and slowing LNA release which is especially important for gene therapy of mitochondrial diseases.

### Biocompatibility and mitochondrial targeting

**Fig. 4:**
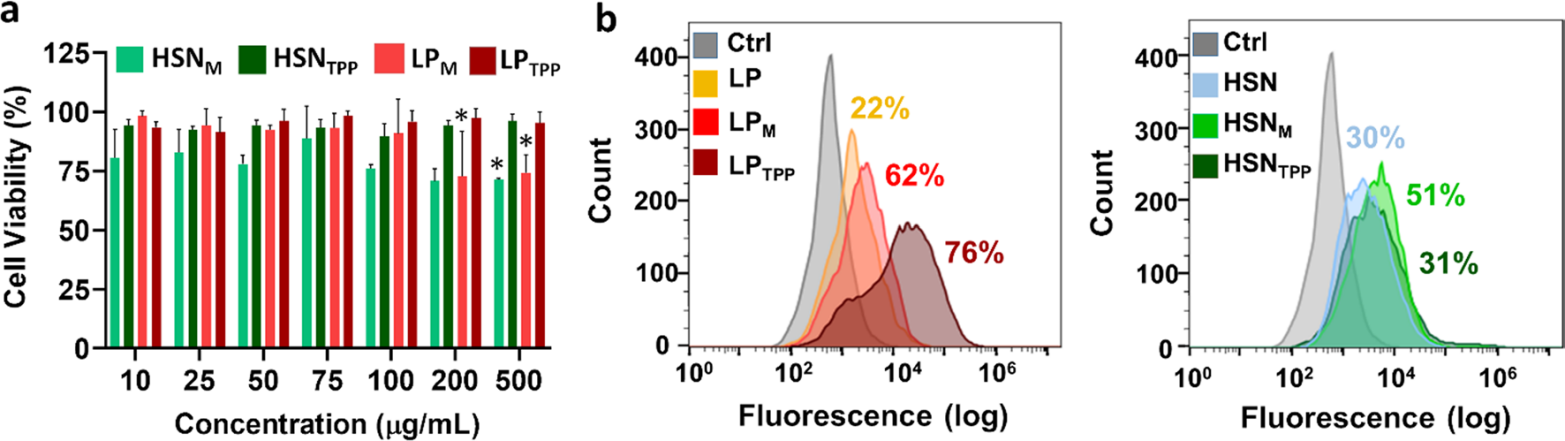
MABs metabolic activity and uptake of bare, TPP, and MITO-Porter functionalized NPs in the MABs cell line. **a** MTS assay of MABs after treatment for 24 h with LP_TPP_ (dark blue), HSN_TPP_ (light blue), LP_M_ (purple), and HSN_M_ (pink) at concentrations from 10 to 500 μg/mL. Viability is expressed as a percentage of unlabeled cell viability. Error bars are derived from the SD of biological triplicates. The statistical significance of cytotoxicity of each functionalized NP is determined compared to 10 μg/mL NP where * = p < 0.001. **b** MABs internalization of LP (red), LP_M_ (purple), LP_TPP_ (dark blue), HSN (green), HSN_M_ (pink), and HSN_TPP_ (light blue) after incubation for 24 h as determined by flow cytometry where left = LP and right = HSN

The biocompatibility and intracellular uptake of MITO-Porter or TPP functionalized HSN and LP was assessed in MABs with an 80% m.3243A > G mutation load derived from an MM-patient, as potential target cells for mitochondrial delivery of oligonucleotides. MABs were exposed for 24 h to 10–500 μg/mL LP_TPP_, HSN_TPP_, LP_M,_ and HSN_M,_ and metabolic activity was assessed using the MTS assay. MABs metabolism was not affected upon exposure up to 100 μg/mL for any of the NPs tested. Concentrations above 200 μg/mL LP_M_ resulted in decreased cell metabolism (*p* < 0.001, Fig. 4a). Next, flow cytometry was used to assess intracellular uptake as a function of MITO-Porter and TPP surface functionalization. MITO-Porter and TPP functionalization significantly increased cell uptake compared to un-functionalized HSN and LP (Fig. 4b). Specifically, 21.7 ± 2.3% of cells had taken up LP while 61.8 ± 6.1% and 75.7 ± 2.6% of MABs had internalized LP_M_ or LP_TPP_, respectively (*p* < 0.0001, Fig. 4b- left). A similar trend was observed for HSN; 29.5 ± 0.7% of MABs took up HSN and 50.7 ± 3.2% contained HSN_M._ However, 31.4 ± 9.1% of MABs had internalized HSN_TPP,_ which was indistinct from HSN uptake efficiency (*p* < 0.001, Fig. 4b- right). Fluorescence microscopy images revealed that HSN were mostly observed as extracellular intracellular aggregates, while some LP were observed in the cytoplasm (Figure S13). On the other hand, high intensities of MITO-Porter and TPP functionalized NP were observed in intracellular locations.

**Fig. 5:**
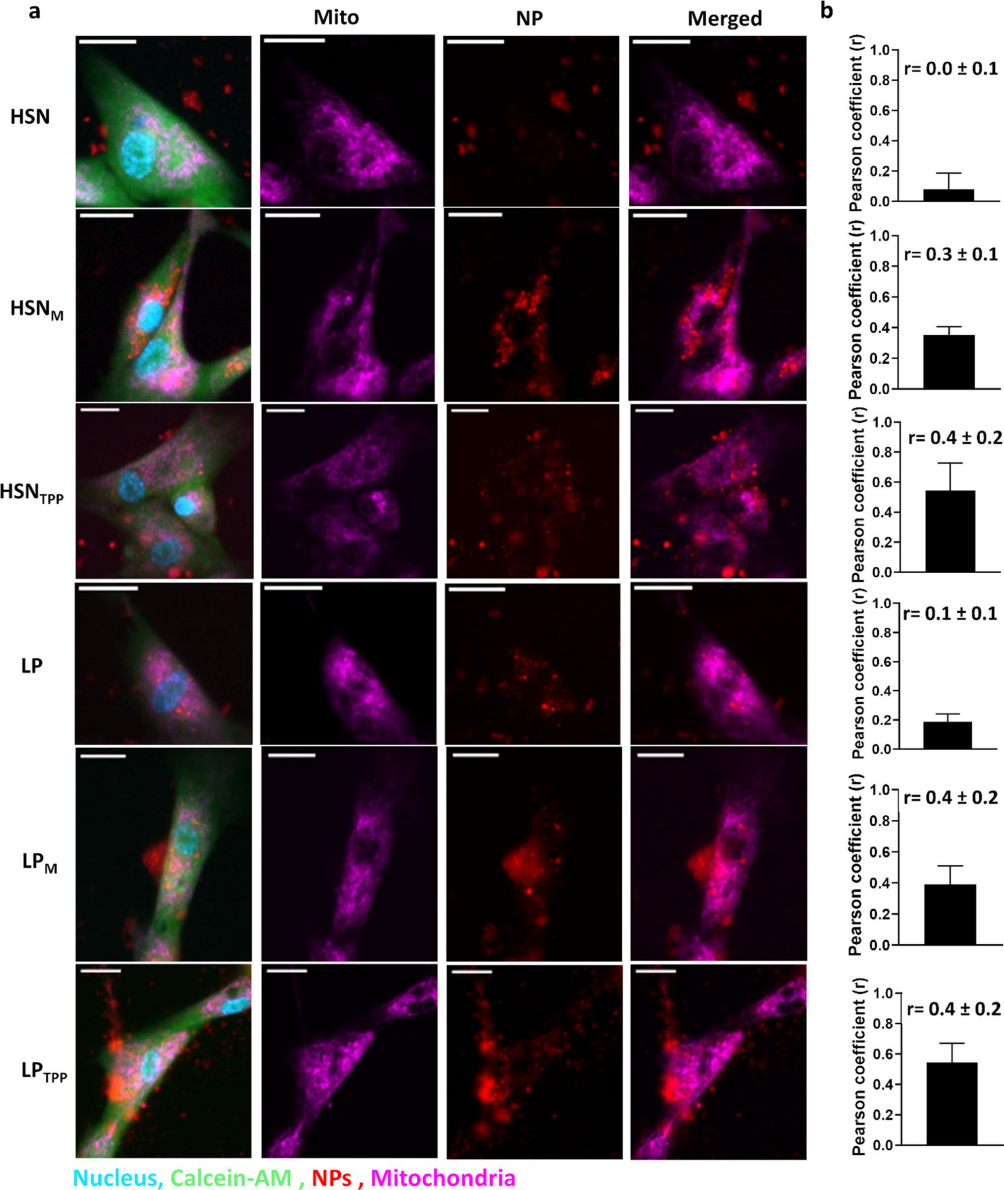
Mitochondrial targeting efficiency of MITO-Porter and TPP functionalized HSN and LPs by co-localization using fluorescent microscopy and TEM. Functionalized NP at 100 µg/mL were incubated with MABs for 24 h. **a** Representative fluorescence microscopy images of MABs stained with calcein-AM, hoechst and mitotracker where localization of mitochondria (purple) and NP (red) is shown in merged images. Scale bars are 25 µm. **b** Pearson’s correlation coefficient (r) comparing the fluorescence of mitotracker to NP in 6 microscopy images

**Fig. 6:**
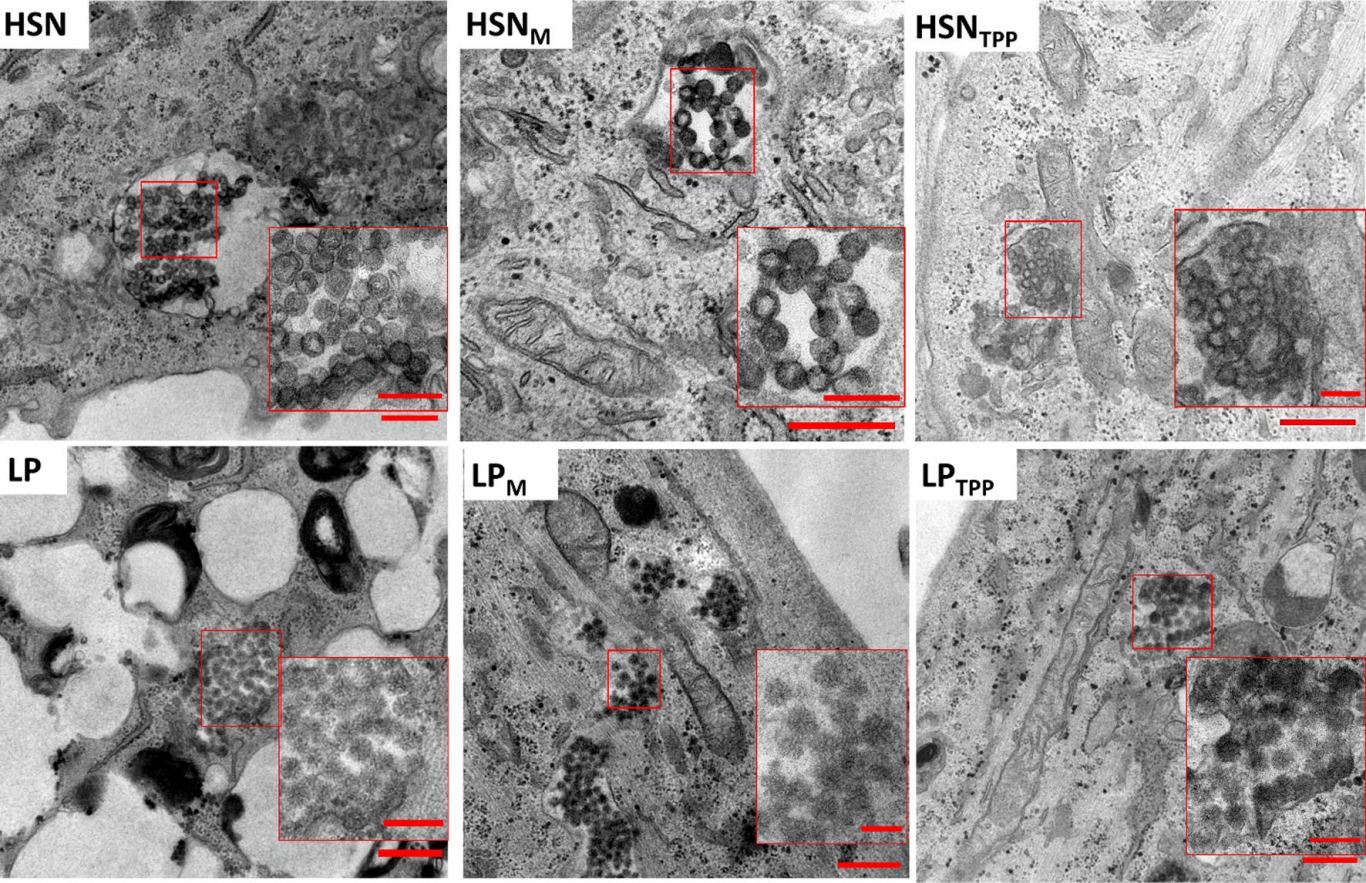
TEM images of fixed, sectioned and stained MABs where unmodified HSN and LP were not often observed near mitochondria while LP_M_ and HSN_M_ were proximal to mitochondria and LP_TPP_ and HSN_TPP_ could be endocytosed into mitochondria. Scale bars are 500 nm in main images and 200 nm in inserts

The mitochondrial targeting ability of functionalized HSN and LP was assessed by fluorescence microscopy using MitoTracker deep red and TEM imaging after 24 h exposure (Fig. 5). Overlap of the MitoTracker channel with RITC-incorporated NP was analyzed qualitatively in merged channels (Fig. 5a) while mitochondrial co-localization was quantified by Pearson’s correlation coefficient (r) analysis of 6 microscopy images (Fig. 5b). Pearson’s correlation coefficient (r) is a measurement of linear correlation from -1 (negative) to 1 (positive) which in this case refers to the spatial co-occurrence of NP fluorescence at 565 nm with mitochondria fluorescence at 647 nm (Fig. 5b) [57]. We observed no correlation in the localization of bare HSNs with mitochondria since r was found to be 0.0 ± 0.1, while for bare LP a slight positive correlation was observed; 0.1 ± 0.1. For TPP and MITO-Porter modified HSN there was a significant increase in mitochondrial co-localization to 0.4 ± 0.2 or 0.3 ± 0.1, respectively (Fig. 5b). While for LP, MITO-Porter and TPP functionalization increased Pearson’s coefficient to the same extent (0.4 ± 0.2, Fig. 5b). Mitochondrial localization was also assessed by TEM imaging after 24 h exposure. We observed that HSN and LP without surface modification did not appear near mitochondria, while TPP and MITO-Porter functionalized NP were mostly observed at mitochondria proximal locations (Fig. 6). Mitochondrial uptake of TPP functionalized NP could be observed for HSN_TPP_ (Fig. 6). Further, LP_TPP_ and HSN_TPP_ could escape endosomes while LP_M_ and HSN_M_ remained mainly within endosomes (Figure S14).

Here we showed that TPP and MITO-Porter functionalized LP and HSN nanoparticles are biocompatible, and show increased cell-internalization and mitochondria targeting ability in patient derived MABs compared to non-functionalized HSN and LP. All NP formulations were non-toxic up to high concentrations of 100 μg/mL (Fig. 4a). For MITO-porter functionalized NP, we observed mild toxicity at concentrations above 100 μg/mL. Similar results were obtained by Kawamura et al. and is likely the effect of octa-arginine, which is known to become toxic at high concentrations [58, 59]. We also showed that surface functionalization of HSN and LP with either TPP or MITO-Porter led to enhanced cell uptake compared to non-functionalized HSN and LP. It has been well documented that the surface properties of NP have a drastic effect on the degree of cell uptake [60, 61]. Specifically, it is known that positively charged, lipophilic NP surfaces lead to high cell uptake [25, 62]. Thus, it is unsurprising that NP modification with positively charged lipid bilayer MITO-Porter and positive, phenyl-rich TPP increases cell uptake. However, it is unclear why we observed a difference between TPP-functionalized HSN and LP.

Surface functionalization with TPP and MITO-Porter also led to significantly increased mitochondrial localization compared to un-functionalized HSN and LP. Mitochondrial co-localization was quantified by Pearson’s correlation coefficient (r), which is deemed more accurate than other approaches since the calculation is based on mean intensity derivation [57]. We found that HSN were randomly distributed (r ~ 0.0) while LP were slightly co-localized with mitochondria (r ~ 0.1), probably a result of the higher surface charge of LP. High surface charge can lead to mitochondrial targeting as a result of the negative membrane potential of mitochondria [63]. However, when HSN and LP were functionalized with TPP and MITO-Porter, Pearson’s coefficient significantly increased (r ~ 0.3–0.4). Although it has been suggested that only r > 0.5 indicates significant colocalization [57], artificially high r values can be easily created if background pixels are not eliminated [64], which is the case for most mitochondrial co-localization studies [65–67]. Here, we set color thresholds to the cell perimeter based on calcein, eliminating background. Pearson’s analysis also revealed that MITO-Porter functionalized HSN targeted different mitochondria within cells evenly; a feature important for homogeneous mitochondrial drug delivery.

TEM imaging confirmed that MITO-Porter functionalized NP were internalized, but mostly trapped in endosomes (Figure S14). It is known that cell-penetrating peptide (CPP) octa-arginine promotes micropinocytosis leading to entrapment in macropinosomes [68]. However, CPP also stimulates escape from macropinosomes by membrane fusion [68–70]. As such, our observation indicates that endosomal escape likely occurs 24 h after cell uptake. In contrast, we showed that TPP functionalized NP were capable of endosomal escape within 24 h (Figure S14). TPP-mediated endosomal escape has been previously demonstrated and is likely a result of the proton sponge effect where positively charged intra-endosomal TPP causes an influx of chloride ions that leads to osmotic swelling and eventual endosomal rupture [71]. Our data indicate that TPP-mediated cell uptake and endosomal escape happens faster in comparison to MITO-Porter, which is possibly due to TPP uptake occurring by endocytosis rather than micropinocytosis [72]. Nevertheless, both MITO-Porter and TPP functionalized NP were located proximal to mitochondria as shown by TEM imaging. Mitochondrial internalization of NP has rarely been visualized however, TEM analysis is critical for making adequate conclusions about the intracellular location, status of degradation, endosomal entrapment and mitochondrial internalization of NP. With use of TEM imaging, we show that our HSN were capable of endosomal escape, located proximal to mitochondria and were not degraded prior to mitochondrial uptake enabling mitochondrial targeted gene delivery. Overall, we demonstrated that HSN and LP surface functionalized with MITO-Porter and TPP were biocompatible, internalized into MABs and showed significant mitochondrial targeting ability.

## Conclusions

Here we developed oligonucleotide-encapsulated HSN and LP capable of controlled, targeted oligonucleotide delivery to mitochondria. We are the first to demonstrate RITC-incorporated LP containing multiple functionalizations throughout their core structure. Further, we describe the first HSN with both RITC and BTES incorporated in the silica matrix and with core-entrapped oligonucleotides that release in intracellular conditions by HSN degradation. Our stable HSN system eliminates the need for tedious loading and gating modifications to exert control over oligonucleotide release. In addition, LNA incorporated HSN and LP were successfully functionalized with TPP and MITO-Porter and displayed a wide range of LNA release profiles from slow, degradation-specific LNA release of HSN(LNA) to fast, non-specific LNA release for LP(LNA). In particular, MITO-Porter functionalization of HSN(LNA) and LP(LNA) enabled intracellular responsive, delayed LNA release and represents a new method to tailor release kinetics for an array of different cell types and therapy models. Further, TPP and MITO-Porter functionalized HSN and LP were biocompatible, increased internalization in MABs, and co-localized with mitochondria as revealed by TEM, fluorescence microscopy, and flow cytometry. Although the focus of this study was mitochondrial delivery of short oligonucleotides, due to the modifiable pore structure, our HSN and LP systems can be easily adapted to deliver nucleases, proteins, and large oligonucleotides. Further, the surface functionalization potential of HSN and LP enables the attachment of different therapeutic, targeting, and imaging agents. Overall, our fluorescent, mitochondria-targeted HSN and LP constructs hold the potential to deliver (large) therapeutics to diseased mitochondria which currently afflict thousands of people worldwide, but could also be optimized to treat countless other genetic pathologies.

### Supplementary Information

Additional file1 (DOCX 4755 KB)

## Data Availability

All the data generated or analyzed during this study are incorporated in this article [and supplementary information file].

## References

[1] Marí M, Morales A, Colell A, García-Ruiz C, Fernández-Checa JC (2009). Mitochondrial Glutathione, a Key Survival Antioxidant,". Antioxid Redox Signal.

[2] Mandrup OA, Lykkemark S, Kristensen P (2017). "Targeting of phage particles towards endothelial cells by antibodies selected through a multi-parameter selection strategy," (in eng). Sci rep.

[3] Bacman SR (2018). MitoTALEN reduces mutant mtDNA load and restores tRNAAla levels in a mouse model of heteroplasmic mtDNA mutation. Nat Med.

[4] Yamada Y, Harashima H (2023) RNA Delivery to Mitochondria (in eng), Handb Exp Pharmacol 10.1007/164_2023_650 37017791

[5] Shirley JL, de Jong YP, Terhorst C, Herzog RW (2020). Immune responses to viral gene therapy vectors. Mol Ther.

[6] Soldatov VO (2022). Current advances in gene therapy of mitochondrial diseases (in eng). J Transl Med.

[7] Zolnik BS, González-Fernández AF, Sadrieh N, Dobrovolskaia MA (2010). Minireview: nanoparticles and the immune system. Endocrinology.

[8] Wang K, Kievit FM, Zhang M (2016). Nanoparticles for cancer gene therapy: recent advances, challenges, and strategies. Pharmacol Res.

[9] Bavnhøj CG, Knopp MM, Madsen CM, Löbmann K (2019). The role interplay between mesoporous silica pore volume and surface area and their effect on drug loading capacity. Int J Pharm: X.

[10] Ding B (2019). MnFe2O4-decorated large-pore mesoporous silica-coated upconversion nanoparticles for near-infrared light-induced and O_2_ self-sufficient photodynamic therapy. Nanoscale.

[11] Hartono SB (2014). Synthesis of multi-functional large pore mesoporous silica nanoparticles as gene carriers. Nanotechnol.

[12] Kwon D (2017). "Extra-large pore mesoporous silica nanoparticles for directing in vivo M2 macrophage polarization by delivering IL-4," (in eng). Nano Lett.

[13] Rahmani S (2019). Large pore mesoporous silica and organosilica nanoparticles for pepstatin a delivery in breast cancer cells, (in eng). Molecules.

[14] Hosseinpour S, Cao Y, Liu J, Xu C, Walsh LJ (2021). Efficient transfection and long-term stability of rno-miRNA-26a-5p for osteogenic differentiation by large pore sized mesoporous silica nanoparticles. J Mater Chem B.

[15] Zhang X, Zeng D, Li N, Jiang X, Liu C, Li Y (2016). Large-pore mesoporous Ca–Si-based bioceramics with high in vitro bioactivity and protein adsorption capability for bone tissue regeneration. J Mater Chem B.

[16] Xiong L, Du X, Kleitz F, Qiao SZ (2015). "Cancer-cell-specific nuclear-targeted drug delivery by dual-ligand-modified mesoporous silica nanoparticles," (in eng). Small.

[17] Ibragimova AR (2021). "Mitochondria-targeted mesoporous silica nanoparticles noncovalently modified with triphenylphosphonium cation: Physicochemical characteristics, cytotoxicity and intracellular uptake," (in eng). Int J Pharm.

[18] Chang F-P, Chen Y-P, Mou C-Y (2014). Intracellular implantation of enzymes in hollow silica nanospheres for protein therapy: cascade system of superoxide dismutase and catalase. Small.

[19] Chang F-P, Hung Y, Chang J-H, Lin C-H, Mou C-Y (2014). Enzyme encapsulated hollow silica nanospheres for intracellular biocatalysis. ACS Appl Mater Interfaces.

[20] Yamada Y (2008). "MITO-Porter: a liposome-based carrier system for delivery of macromolecules into mitochondria via membrane fusion," (in eng). Biochim Biophys Acta.

[21] Tonlorenzi R, Dellavalle A, Schnapp E, Cossu G, Sampaolesi M (2007). "Isolation and characterization of mesoangioblasts from mouse, dog, and human tissues," (in eng). Curr Protoc Stem Cell Biol.

[22] van Tienen F (2019). Healthy, mtDNA-mutation free mesoangioblasts from mtDNA patients qualify for autologous therapy. Stem Cell Res Ther.

[23] Jatupaiboon N (2015). A facile microemulsion template route for producing hollow silica nanospheres as imaging agents and drug nanocarriers. J Mater Chem B.

[24] Guo Z (2020). Design of dendritic large-pore mesoporous silica nanoparticles with controlled structure and formation mechanism in dual-templating strategy, (in eng). ACS Appl Mater Interfaces.

[25] Rosenbrand R (2018). Lipid surface modifications increase mesoporous silica nanoparticle labeling properties in mesenchymal stem cells, (in eng). Int J Nanomedicine.

[26] Trayford C (2022). Mesoporous silica-coated gold nanoparticles for multimodal imaging and reactive oxygen species sensing of stem cells. ACS Appl Nano Mater.

[27] Costa P, Sousa Lobo JM (2001). Modeling and comparison of dissolution profiles. Eur J Pharm Sci.

[28] Lin C-H, Chang J-H, Yeh Y-Q, Wu S-H, Liu Y-H, Mou C-Y (2015). Formation of hollow silica nanospheres by reverse microemulsion. Nanoscale.

[29] Fan W (2019). Generic synthesis of small-sized hollow mesoporous organosilica nanoparticles for oxygen-independent X-ray-activated synergistic therapy. Nat Comm.

[30] Huang P (2017). Molecularly organic/inorganic hybrid hollow mesoporous organosilica nanocapsules with tumor-specific biodegradability and enhanced chemotherapeutic functionality. Biomaterials.

[31] Guo Z (2020). Design of dendritic large-pore mesoporous silica nanoparticles with controlled structure and formation mechanism in dual-templating strategy. ACS Appl Mater Interfaces.

[32] Gao F, Botella P, Corma A, Blesa J, Dong L (2009). Monodispersed mesoporous silica nanoparticles with very large pores for enhanced adsorption and release of DNA. J Phys Chem B.

[33] Wu M (2015). Large-pore ultrasmall mesoporous organosilica nanoparticles: micelle/precursor co-templating assembly and nuclear-targeted gene delivery. Adv Mater.

[34] Guo K, Zhao X, Dai X, Zhao N, Xu F-J (2019). Organic/inorganic nanohybrids as multifunctional gene delivery systems. J Gene Med.

[35] Zhan Z (2017). Improved gene transfer with functionalized hollow mesoporous silica nanoparticles of reduced cytotoxicity. Materials.

[36] Lin X (2018). Photo-responsive hollow silica nanoparticles for light-triggered genetic and photodynamic synergistic therapy. Acta Biomater.

[37] Ma X, Zhao Y, Ng KW, Zhao Y (2013). Integrated hollow mesoporous silica nanoparticles for target drug/siRNA co-delivery. Chem Eur J.

[38] Kulkarni CA (2021). A novel triphenylphosphonium carrier to target mitochondria without uncoupling oxidative phosphorylation. J Med Chem.

[39] Durfee PN (2016). Mesoporous silica nanoparticle-supported lipid bilayers (protocells) for active targeting and delivery to individual leukemia cells. ACS Nano.

[40] Cauda V (2010). "Colchicine-loaded lipid bilayer-coated 50 nm mesoporous nanoparticles efficiently induce microtubule depolymerization upon cell uptake," (in eng). Nano Lett.

[41] Chaiyana W, Phongpradist R, Leelapornpisid P, Anuchapreeda S (2014). Microemulsion-based hydrogel for topical delivery of indomethacin. Int J Pharm Pharm Sci.

[42] Solberg SM, Landry CC (2006). Adsorption of DNA into mesoporous silica. J Phys Chem B.

[43] Niu D (2014). Monodispersed and ordered large-pore mesoporous silica nanospheres with tunable pore structure for magnetic functionalization and gene delivery. Adv Mater.

[44] Wang T, Larcher LM, Ma L, Veedu RN (2018). Systematic screening of commonly used commercial transfection reagents towards efficient transfection of single-stranded oligonucleotides (in eng). Molecules.

[45] Blechinger J, Bauer AT, Torrano AA, Gorzelanny C, Bräuchle C, Schneider SW (2013). Uptake kinetics and nanotoxicity of silica nanoparticles are cell type dependent. Small.

[46] Fisichella M (2010). "Uptake of functionalized mesoporous silica nanoparticles by human cancer cells," (in eng). J Nanosci Nanotechnol.

[47] Chen L (2018). The toxicity of silica nanoparticles to the immune system. Nanomedicine.

[48] Chen Q (2020). Acidity and glutathione dual-responsive polydopamine-coated organic-inorganic hybrid hollow mesoporous silica nanoparticles for controlled drug delivery. ChemMedChem.

[49] Wang D (2014). Fabrication of single-hole glutathione-responsive degradable hollow silica nanoparticles for drug delivery. ACS Appl Mater Interfaces.

[50] Li Z (2020). Nanoparticle depots for controlled and sustained gene delivery. J Control Release.

[51] Diaz F, Moraes CT (2008). "Mitochondrial biogenesis and turnover," (in eng). Cell Calcium.

[52] Xiong L, Bi J, Tang Y, Qiao S-Z (2016). Magnetic core-shell silica nanoparticles with large radial mesopores for siRNA delivery. Small.

[53] Mudakavi RJ, Raichur AM, Chakravortty D (2014). Lipid coated mesoporous silica nanoparticles as an oral delivery system for targeting and treatment of intravacuolar Salmonella infections. RSC Adv.

[54] Amin MU (2021). Enhanced efficacy and drug delivery with lipid coated mesoporous silica nanoparticles in cancer therapy. Eur J Pharm Biopharm.

[55] LaBauve AE (2018). Lipid-coated mesoporous silica nanoparticles for the delivery of the ML336 antiviral to inhibit encephalitic alphavirus infection. Sci Rep.

[56] Nakamura T, Kuroi M, Harashima H (2015). Influence of endosomal escape and degradation of α-galactosylceramide loaded liposomes on CD1d antigen presentation. Mol Pharm.

[57] Adler J, Parmryd I (2010). Quantifying colocalization by correlation: The Pearson correlation coefficient is superior to the Mander's overlap coefficient. Cytometry A.

[58] Grogg M (2018). Cell penetration, herbicidal activity, and in-vivo-toxicity of oligo-arginine derivatives and of novel guanidinium-rich compounds derived from the biopolymer cyanophycin. Helv Chim Acta.

[59] Kawamura E, Yamada Y, Harashima H (2013). Mitochondrial targeting functional peptides as potential devices for the mitochondrial delivery of a DF-MITO-Porter. Mitochondrion.

[60] Encinas N, Angulo M, Astorga C, Colilla M, Izquierdo-Barba I, Vallet-Regí M (2019). Mixed-charge pseudo-zwitterionic mesoporous silica nanoparticles with low-fouling and reduced cell uptake properties. Acta Biomater.

[61] Shahabi S (2015). Enhancing cellular uptake and doxorubicin delivery of mesoporous silica nanoparticles via surface functionalization: effects of serum. ACS Appl Mater Interfaces.

[62] Kurtz-Chalot A (2017). Impact of silica nanoparticle surface chemistry on protein corona formation and consequential interactions with biological cells. Mater Sci Eng C.

[63] Zorova LD (2018). Mitochondrial membrane potential, (in eng). Anal Biochem.

[64] Barlow AL, MacLeod A, Noppen S, Sanderson J, Guérin CJ (2010). Colocalization analysis in fluorescence micrographs: verification of a more accurate calculation of pearson's correlation coefficient. Microsc Microanal.

[65] Qu G, Jiang T, Liu T, Ma X (2022). Multifunctional host polymers assist au nanoclusters achieve high quantum yield and mitochondrial imaging. ACS Appl Mater Interfaces.

[66] Marrache S, Dhar S (2012). Engineering of blended nanoparticle platform for delivery of mitochondria-acting therapeutics. Proc Natl Acad Sci.

[67] Ahn J, Lee B, Choi Y, Jin H, Lim NY, Park J, Kim JH, Bae J, Jung JH (2018). Non-peptidic guanidinium-functionalized silica nanoparticles as selective mitochondria-targeting drug nanocarriers. J Mater Chem B.

[68] El-Sayed A, Khalil IA, Kogure K, Futaki S, Harashima H (2008). Octaarginine- and octalysine-modified nanoparticles have different modes of endosomal escape*. J Biol Chem.

[69] Yamada Y, Furukawa R, Yasuzaki Y, Harashima H (2011). "Dual function MITO-Porter, a nano carrier integrating both efficient cytoplasmic delivery and mitochondrial macromolecule delivery," (in eng). Mol Ther.

[70] Smith SA, Selby LI, Johnston APR, Such GK (2019). "The endosomal escape of nanoparticles: toward more efficient cellular delivery," (in eng). Bioconjug Chem.

[71] Han X (2019). Triphenylphosphonium-modified mitochondria-targeted paclitaxel nanocrystals for overcoming multidrug resistance. Asian J Pharm Sci.

[72] Gonçalves C, Mennesson E, Fuchs R, Gorvel J-P, Midoux P, Pichon C (2004). Macropinocytosis of polyplexes and recycling of plasmid via the clathrin-dependent pathway impair the transfection efficiency of human hepatocarcinoma cells. Mol Ther.

