# Supplementary material for: One-pot, degradable, silica nanocarriers with encapsulated oligonucleotides for mitochondrial specific targeting
Source: Discov Nano. 2023 Dec 21;18(1):161. doi: 10.1186/s11671-023-03926-1 (PMC10739632; doi:10.1186/s11671-023-03926-1)
Supplement: Supplementary file 1 — Additional file1 (DOCX 4755 KB) [file 11671_2023_3926_MOESM1_ESM.docx]

**Supplementary information**

**One-pot, degradable, silica nanocarriers with encapsulated oligonucleotides for mitochondrial specific targeting**

Chloe Trayford^1^, Alissa Wilhalm^1,2^, Pamela Habibovic^1^, Hubert Smeets^2^, Florence van Tienen^2*^ and Sabine van Rijt^1*^

^1^MERLN Institute for Technology-Inspired Regenerative Medicine, Maastricht University, P.O. Box 616, 6200 MD Maastricht, the Netherlands

^2^Department of Toxicogenomics, Maastricht University, PO Box 616
6200 MD, Maastricht, The Netherlands

* Shared last and corresponding authors:

Sabine van Rijt, PhD

Associate Professor

Department of Instructive Biomaterials Engineering

MERLN Institute for Technology-Inspired Regenerative Medicine, Maastricht University

E-mail: [s.vanrijt@maastrichtuniversity.nl](mailto:s.vanrijt@maastrichtuniversity.nl)

Florence van Tienen, PhD

Assistant Professor

Department of Toxicogenomics

School for Mental Health and Neuroscience, Maastricht University

E-mail: [florence​.​vantienen​@​​maastricht​university​.​nl](mailto:florence.vantienen@maastrichtuniversity.nl)

*
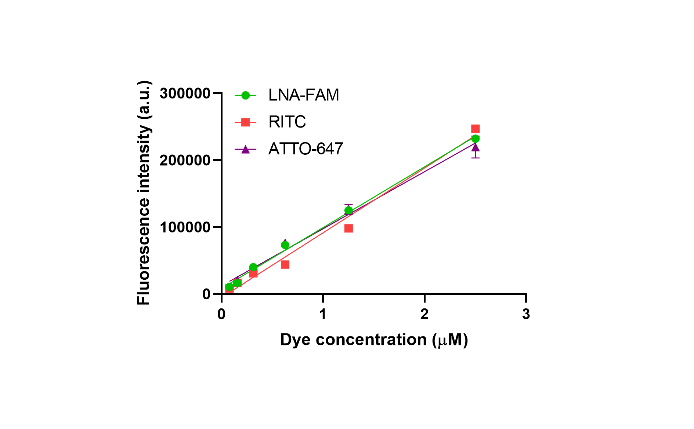
*

**Fig S1**. Rhodamine b isothiocyanate (RITC), Fluorescein locked nucleic acid (LNA-FAM) and ATTO-647 standard curves for the determination of LNA-FAM, RITC and ATTO-647 concentration in HSN and LP.


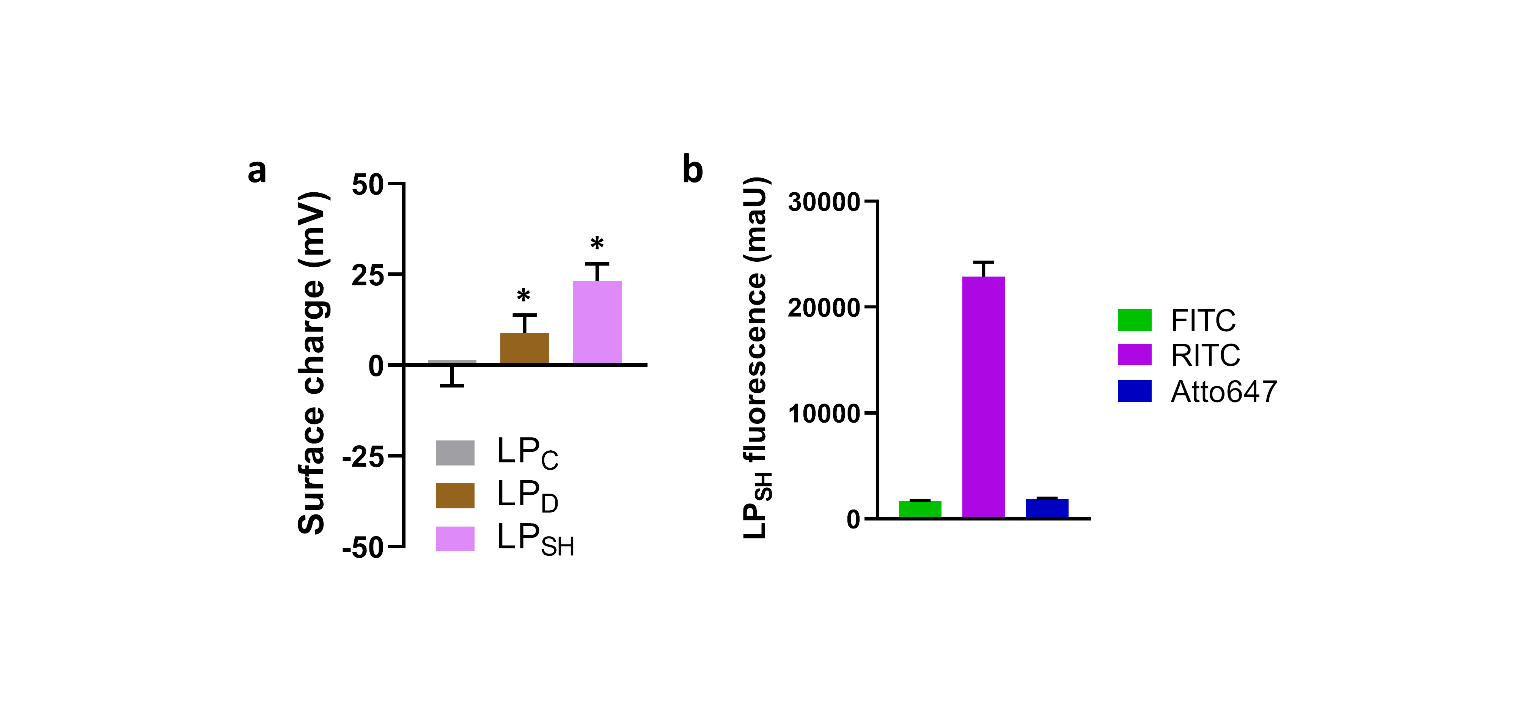


**Fig S2**. a) Surface charge of functionalized LPs where LP_C_ = -0.1 ± 5.6 mV (grey), LP_D_= 8.9 ± 4.9 mV (light brown), LP_SH_ = 23.2 ± 4.7 mV (light purple). Statistical significance of is determined compared to LP_C_ where * = p < 0.0001. b) Fluorescence of LP_SH_ after incubation with amine reactive FITC and thiol reactive ATTO-647-Maleimide.

| *Analog* | *Sequence 5’ to 3’* |
| --- | --- |
| DNA-LNA mixmer | GG[+G] TT[+T] GG[+T] AA[+G] AT[+G] GC[+A] GG |
| PNA | TGG CAG GGC CCG |
| DNA | GGG TTT GGT AAG ATG GCA GG-C3 |

**Table S1**. Oligonucleotide options to block the m.3243A>G mutation and stop amplification. The location of the mutation is indicated in red.


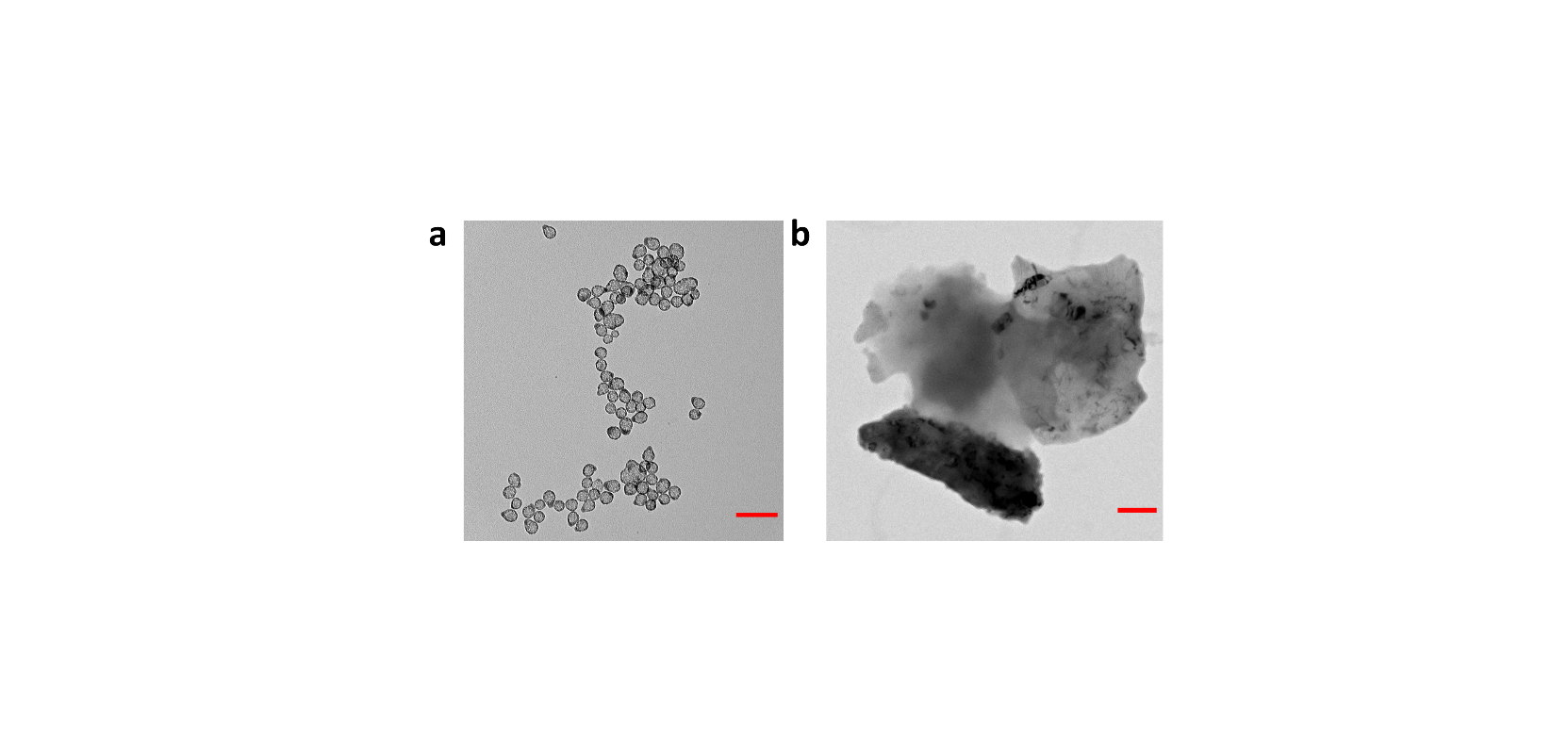


**Fig S3**. TEM images of HSN incorporated with different oligonucleotides showing effects on morphology where a) HSN(DNA) and b) HSN(PNA). Scale bars are 200 nm.

*
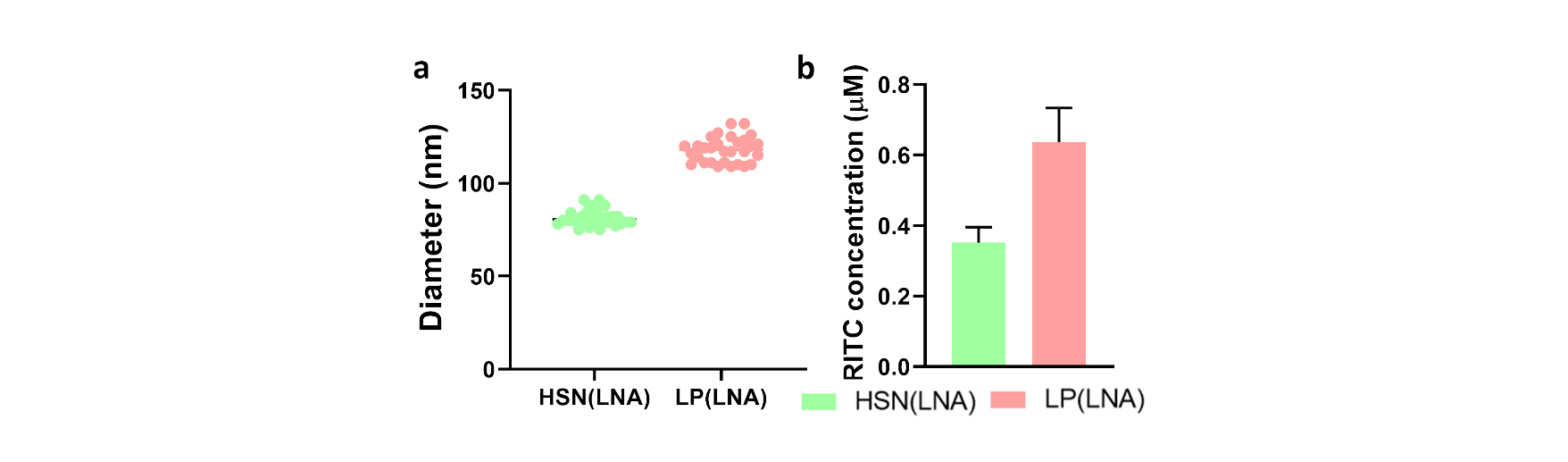
*

**Fig S4**. Size and fluorescence characterization of HSN(LNA). a) Size analysis of 30 particles by ImageJ where HSN (light green)= 82.5 ± 6.3 nm and LP (pink)= 117.9 ± 6.6 nm b) Concentration of RITC in 100 µg of NP as determined by fluorescence analysis where RITC is 0.4 ± 0.04 µM or 0.6 ± 0.08 µM in HSN(LNA) (light green) and LP(LNA) (pink), respectively.

**
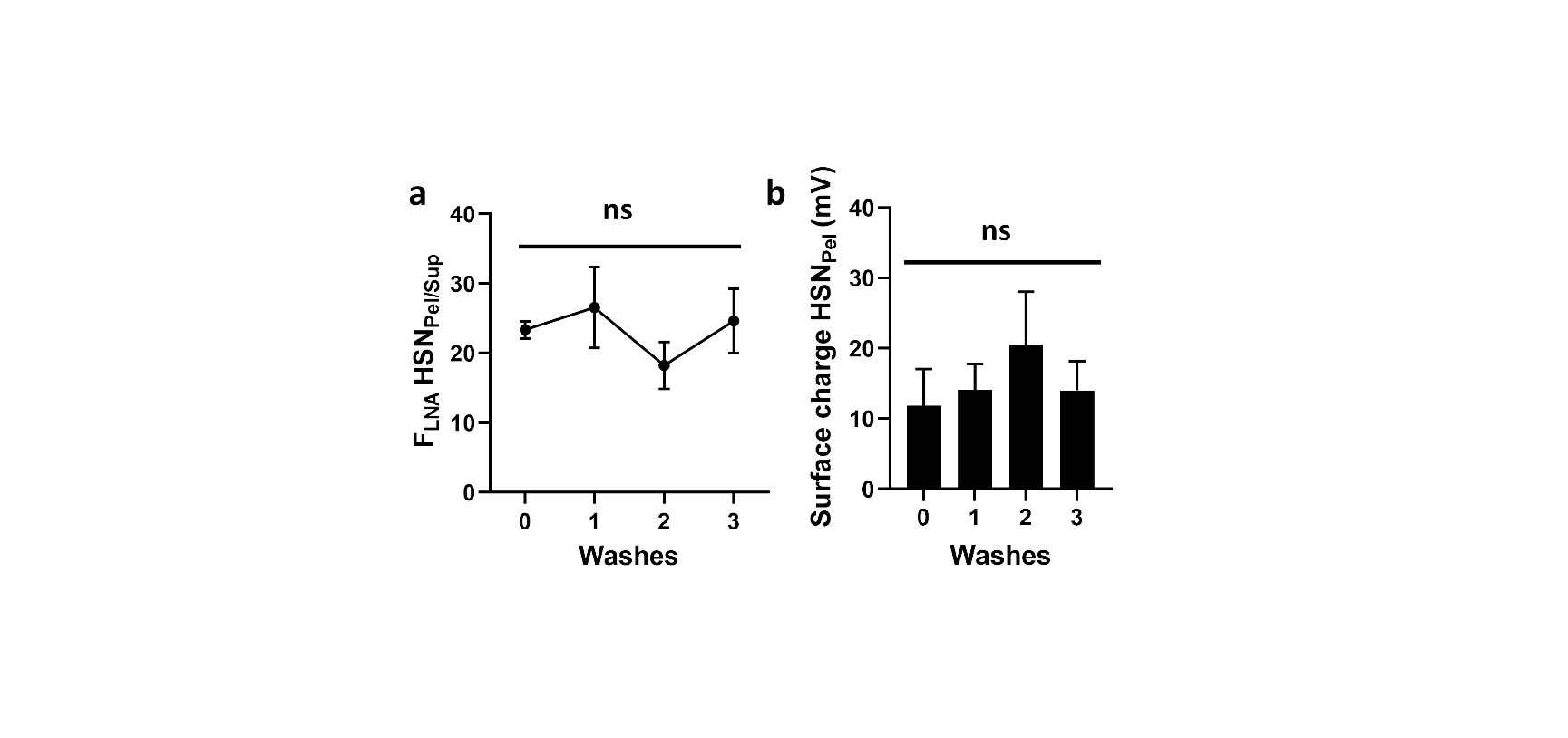
**

**Fig S5**. Incorporation of LNA inside the hollow core of HSN(LNA-FAM). Incorporation was determined by a) ratio of pellet: supernatant LNA-FAM fluorescence in HSN(LNA-FAM) at each of 3 washes with absolute ethanol and b) corresponding surface charges of HSN pellets at each wash. Error bars are derived from technical triplicates.

*
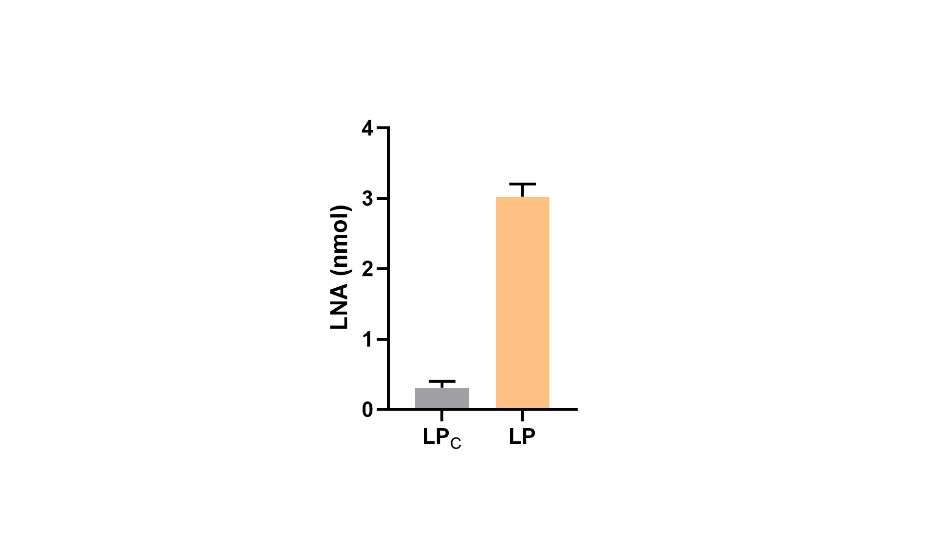
*

**Fig S6**. LNA-FAM content per mg of LP_C_ and LP by UV absorption analysis*.*

*
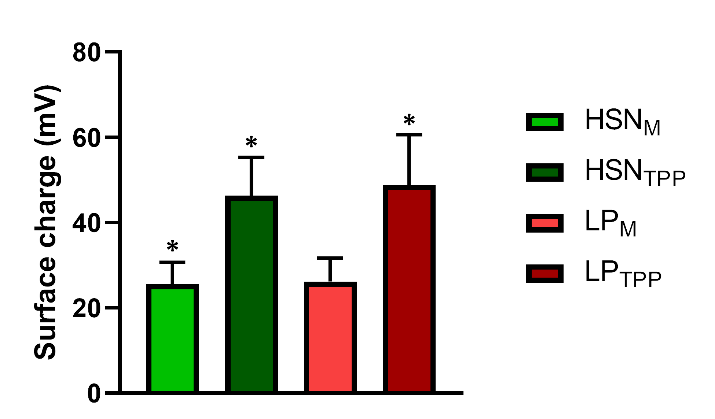
*

**Fig S7**. Zeta potential analysis of MITO-porter and TPP functionalized HSN and LP where HSN_M_=green, HSN_TPP_=dark green, LP_M_= red and LP_TPP_= dark red. MITO-porter and TPP functionalization significantly increased the surface charge of HSN and LP where * = p<0.0001.

*
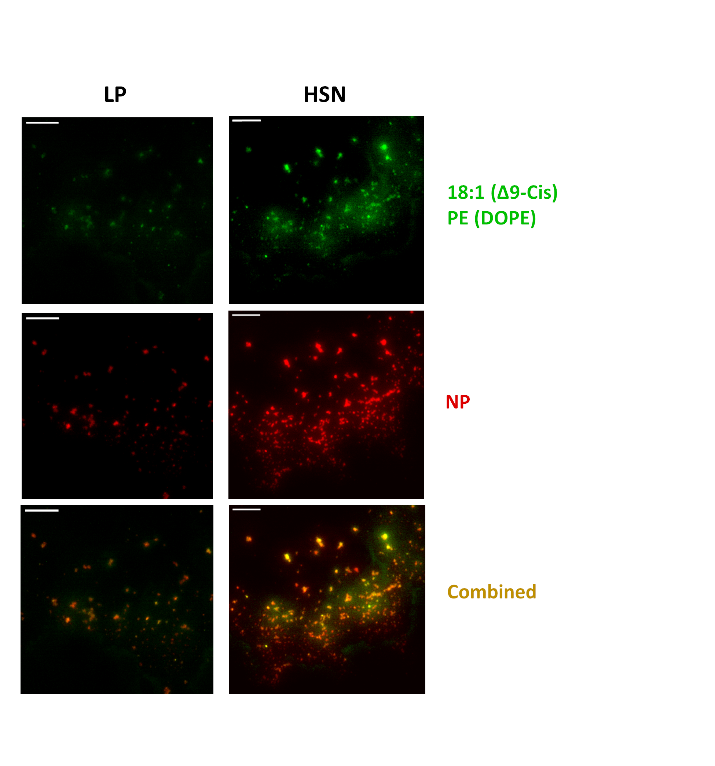
*

**Fig S8**. Fluorescence imaging of fluorescent MITO-porter functionalized HSN and LP showing co-localization of the substituted fluorescein DOPE; 18:1 (Δ9-Cis) PE (DOPE) with RITC NP fluorescence. NP were coated with MITO-porter by hydration of the lipid film, washed three times and deposited on a plasmalysed microscopy cover slip. Where green= 18:1 (Δ9-Cis) PE (DOPE) red= HSN or LP and orange= merged channels. Scale bars are 50 μm.

*
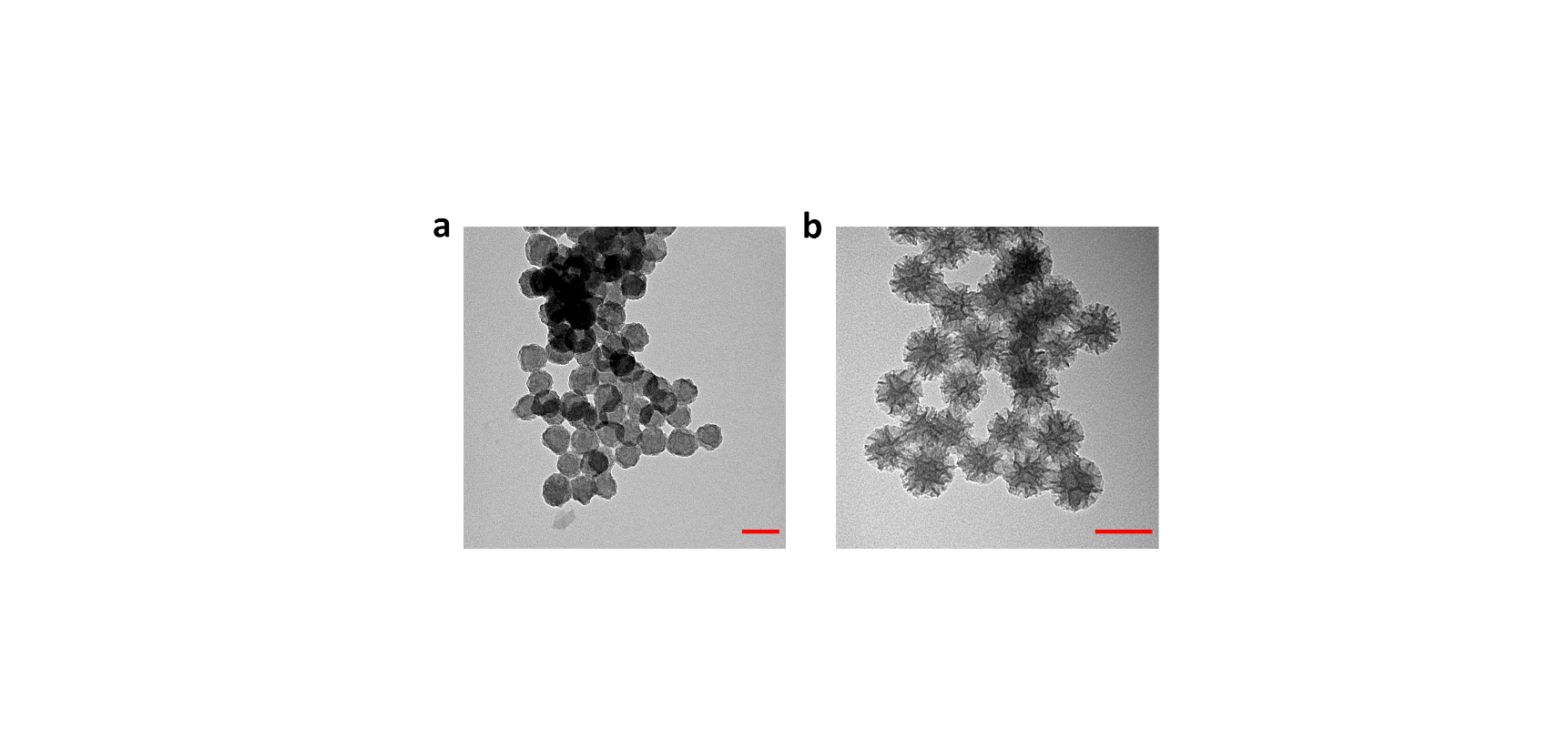
*

**Fig S9**. TEM images of uranyl acetate stained HSN_M_ and LP_M_ where a) HSN_M_ and b) LP_M_. Scale bar is 100nm.


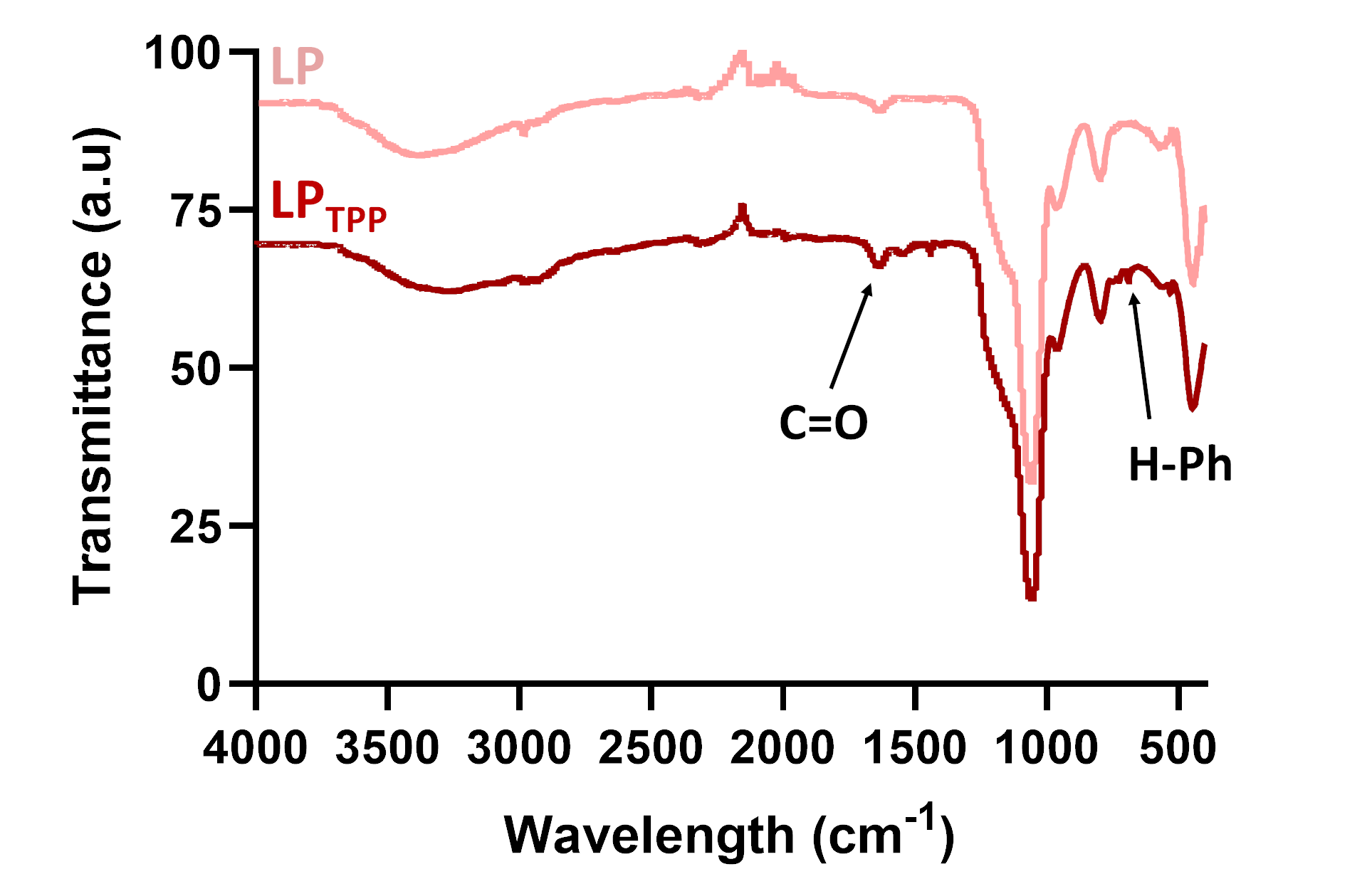


**Fig S10**. FTIR spectrum of LPs and LP_TPP_ to show successful TPP conjugation on the NP surface where LP= pink and LP_TPP_= dark red. Characteristic peaks referring to the amide bonds or phenyl groups of conjugated TPP are indicted by arrows.


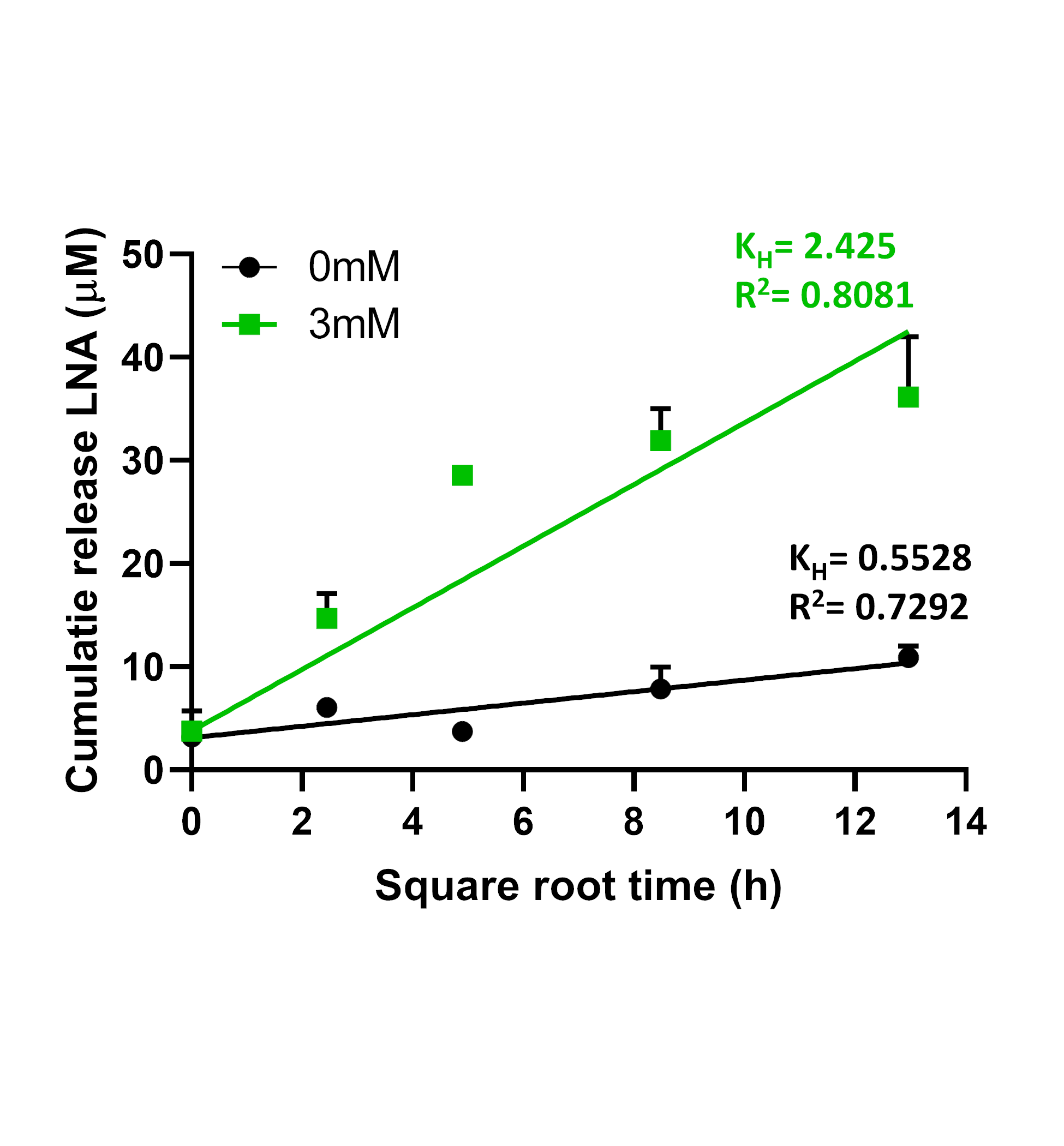


**Fig S11**. LNA release profiles of HSN(LNA)_TPP_ fitted to the Higuchi model. Where HSN(LNA)_TPP_ dispersed in PBS = black and HSN(LNA)_TPP_ dispersed in PBS with 3mM GSH = green. The Higuchi dissolution constant K_H_ and the coefficient of determination R^2^ are given above the fittings.

*
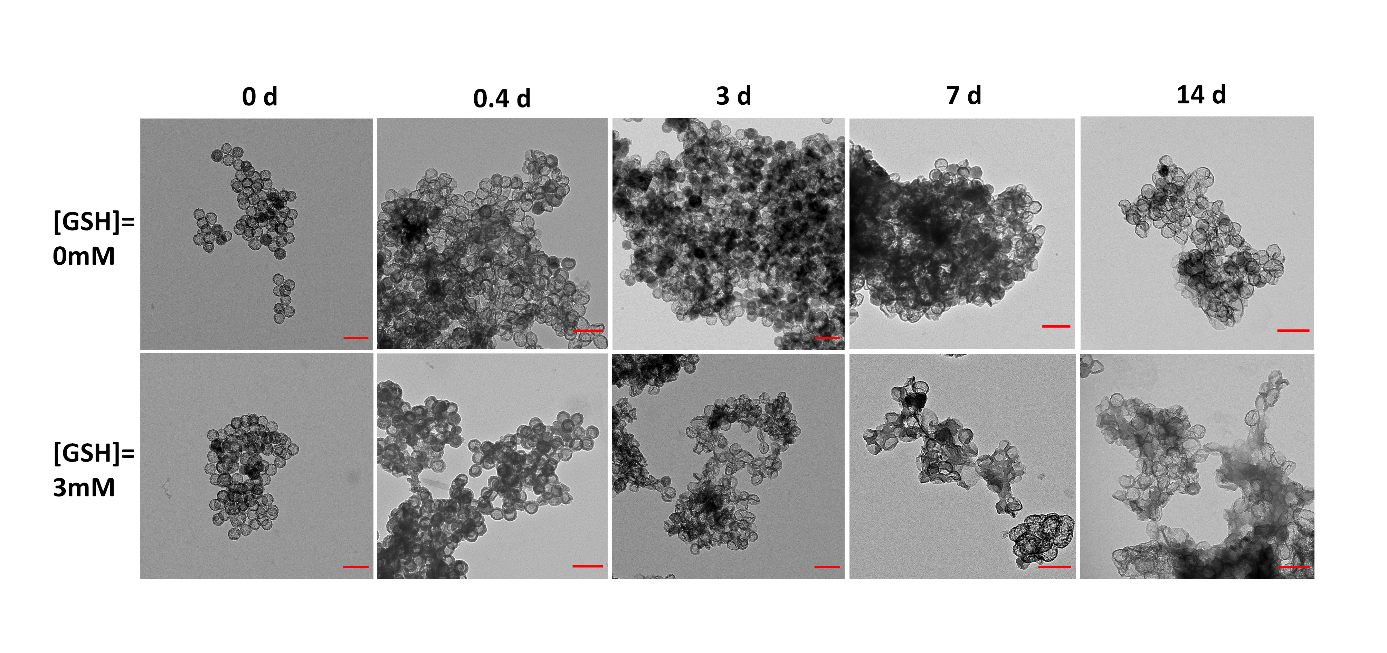
*

**Fig S12.** TEM images showing GSH catalyzed degradation of HSN(LNA)_M_ over a 14 d period. A 50 µL aliquot from a stock of HSN(LNA)_M_ stirred at 37°C in PBS or in PBS with 3 mM GSH was collected and imaged at each timepoint. HSN(LNA)_M_ degradation was only obvious at 7 and 14 d for the 3 mM GSH condition. Scale bars are 200 nm.


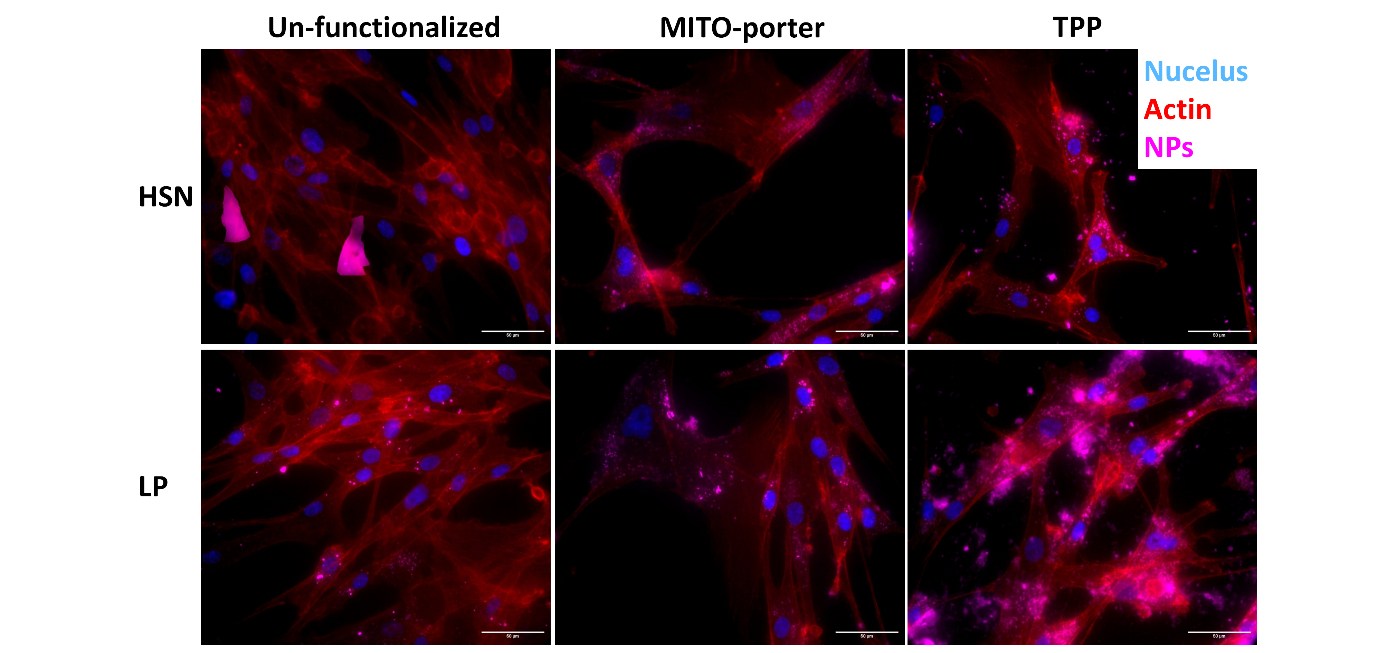
**Fig S13**. Fluorescence imaging of 80% mutated mesangioblast cells (M32) labelled with un-functionalized, MITO-porter and TPP functionalized HSN and LP. Images show intracellular distribution. M32 were exposed to NPs at 100 μg/mL and images were taken 24 h post labelling where blue= nucleus, red= actin and pink= NP. Scale bars are 50 μm.


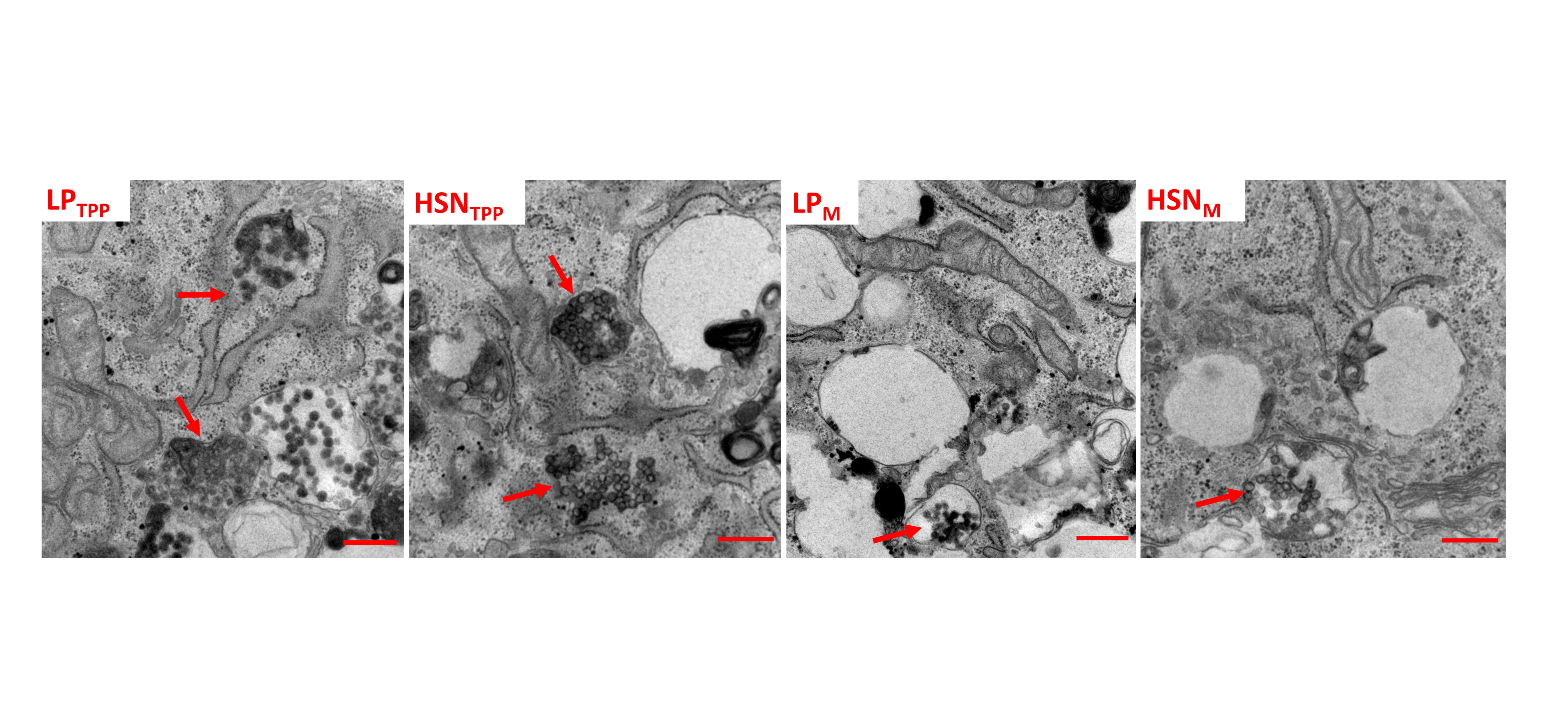


**Fig S14**. TEM images showing endosomal localization of MITO-porter or TPP functionalized HSN and LP. 80% mutated mesangioblasts (M32) were incubated with NP at 100 μg/mL then fixed, sectioned, stained and imaged after 24 h exposure. While LP_TPP_ and HSN_TPP_ were mostly observed to escape endosomes, LP_M_ and HSN_M_ are contained within them. Arrow indicate nanoparticle clusters. Scale bars are 500 nm.
